# Natural products for the treatment of chemotherapy-related cognitive impairment and prospects of nose-to-brain drug delivery

**DOI:** 10.3389/fphar.2024.1292807

**Published:** 2024-01-29

**Authors:** Yu-Qiong He, Can-Can Zhou, Sheng-Gui Jiang, Wen-Qian Lan, Feng Zhang, Xia Tao, Wan-Sheng Chen

**Affiliations:** ^1^ Institute of Chinese Materia Madica, Shanghai University of Traditional Chinese Medicine, Shanghai, China; ^2^ Department of Pharmacy, Changzheng Hospital, Second Military Medical University, Shanghai, China; ^3^ Department of Pharmacy, Shanghai Tenth People’s Hospital, Tongji University School of Medicine, Shanghai, China

**Keywords:** natural products, chemotherapy, cognitive deficits, mechanisms, nose-tobrain-drug-delivery

## Abstract

Chemotherapy-related cognitive deficits (CRCI) as one of the common adverse drug reactions during chemotherapy that manifest as memory, attention, and executive function impairments. However, there are still no effective pharmacological therapies for the treatment of CRCI. Natural compounds have always inspired drug development and numerous natural products have shown potential therapeutic effects on CRCI. Nevertheless, improving the brain targeting of natural compounds in the treatment of CRCI is still a problem to be overcome at present and in the future. Accumulated evidence shows that nose-to-brain drug delivery may be an excellent carrier for natural compounds. Therefore, we reviewed natural products with potential anti-CRCI, focusing on the signaling pathway of these drugs’ anti-CRCI effects, as well as the possibility and prospect of treating CRCI with natural compounds based on nose-to-brain drug delivery in the future. In conclusion, this review provides new insights to further explore natural products in the treatment of CRCI.

## 1 Introduction

Chemotherapy as a standard treatment for cancer has been used since the early 20th century (DeVita and Chu, 2008). In 2020, more than 19 million people were diagnosed with cancer globally and new estimates suggested that there are approximately 50 million survivors 5 years after being diagnosed with cancer (Schagen et al., 2022). Over the past decades, chemotherapy has considerably improved the survival rates of patients with cancer. However, due to a lack of cellular specificity, chemotherapy may have deleterious effects on multiple organs and tissues, with special reference to the central nervous system (CNS), which eventually affects the survivors’ quality of life.

“Chemotherapy-related cognitive deficits (CRCI)” also named “Chemobrain” is described as short/long-term memory impairment characterized by difficulties in attention, learning, working memory, and executive function (Gibson et al., 2019). Cognitive deficits are reported in over 75% of patients who have undergone chemotherapy for cancer, and it persists in 17%–34% of survivors-received chemotherapy (Das et al., 2020). Many scientists have investigated the progression of CRCI in both cross-sectional and longitudinal studies, and have reported that CRCI emerges in survivors of breast cancer, lung cancer, leukemia, lymphoma, nasopharyngeal carcinoma, ovarian cancer, prostate cancer, and testicular cancer (Argyriou et al., 2011; Lv et al., 2020). For example, a longitudinal study conducted in Houston showed that more than 65% of breast cancer patients underwent cognitive impairment shortly after chemotherapy, and almost 61% had persistent cognitive deficits (Wefel et al., 2010). Results from a study on patients with nasopharyngeal carcinoma also demonstrated cognitive impairment in about 25% of patients who received chemotherapy, reflected by remarkably decreased attention, short-term memory, and language abilities (Wang et al., 2020). A cross-sectional study in Norway reported that almost half of the cervical cancer survivors self-reported sustained cognitive impairment (Areklett et al., 2022). Currently, the national cancer institute has considered CRCI as one of the most debilitating side effects of chemotherapy, which largely prevent cancer survivors from resuming their lives (Kumar, 2021).

Although CRCI was initially regarded as a temporary symptom, studies have shown that these symptoms persisted from 1 month to 10 years following treatment (Jim et al., 2012; Koppelmans et al., 2012). Recently, numerous structural and functional neuroimaging studies have confirmed that these functional impairments are related to multiple brain regions, including the frontal lobes, the temporal area, and especially the hippocampus regions (Argyriou et al., 2011). In a prospective magnetic resonance imaging study, McDonald et al. found decreased gray matter density in the frontal, temporal cortices, and cerebellum regions of breast cancer survivors (de Ruiter et al., 2012). Diffusion tensor imaging study also showed that compared with the healthy, the fractional anisotropy values of white matter fibers in the frontal and temporal areas of post-chemotherapy breast cancer patients were significantly decreased, which were significantly associated with attention and processing/psychomotor speed (Deprez et al., 2011). Besides, abnormal resting cerebral vascular density and cerebral blood flow alterations in patients treated with chemotherapy have also been reported (Nudelman et al., 2014; Nudelman et al., 2016). However, concerning the mechanisms of CRCI, the precise pathological mechanisms of the chembrain are still elusive. According to the anti-cancer mechanisms and chemical structure, chemotherapeutic agents are classified as alkylating agents, such as cyclophosphamide (CYP) and methotrexate; antimetabolites, such as 5-fluorouracil (5-FU); and anthracyclines, such as doxorubicin (DOX). Different chemotherapeutics kill cancer cells via various mechanisms. For example, CYP, a representative alkylating agent, induces DNA damage to induced cell apoptosis. Methotrexate and 5-FU interfere with the biosynthesis and functions of DNA or RNA, resulting in cell death; Anti-microtubule agents, such as paclitaxel and docetaxel, can disturb cell division and proliferation (Mounier et al., 2020). Each of these agent kills tumor cells in different ways and may also induce CRCI through different mechanisms, including disturbed neurotransmission, overproduction of free radicals, DNA damage, impaired neurogenesis, as well as glial cell over-activation, and increased neuroinflammation.

Although several western agents including CNS stimulants and anti-dementia drugs are presently being tested in clinical trials, there is no clinically effective drug to prevent or treat CRCI (Karschnia et al., 2019). In human history, herbal formulas and their extracts have been used in the treatment of human diseases due to their high efficacy and low toxicity. Currently, more than one-third of the most popular pharmaceuticals are originated from natural products or their derivatives. Compared with synthetic drugs, natural products are more widely accepted because they are relatively safer and have a better affinity to target proteins or specific biomolecules in humans. In the face of clinical and fundamental research accumulated over the centuries, it is hard to ignore the neuroprotective effects of natural products. A variety of natural products, such as flavonoids, alkaloids, and terpenoids have been recognized as potential therapeutic agents for CNS diseases, including Alzheimer’s disease (AD), Parkinson’s disease (PD), multiple sclerosis, and cerebral ischemia (Dey et al., 2017; Liang et al., 2021). It has been reported that these neuroprotective natural products modulate multiple signaling pathways by directly affecting enzymes, such as kinases, regulatory receptors, and proteins. This broad spectrum of pharmacological and biological activities has made them attractive candidates for the treatment of CRCI.

In this review, we clarified the primary mechanisms of CRCI and summarize the interventions with natural products in protecting against CRCI and the molecular events of the underlying mechanisms. Finally, we highlighted the potential feasibility of natural products-based nose-to-brain drug delivery in treating CRCI. This review provides a new sight into a comprehensive understanding of the prevention of CRCI and potential implications for the protective effects of natural products in chemotherapy patients.

## 2 Mechanisms of chemotherapy-related cognitive impairment

### 2.1 Oxidative stress

**FIGURE 1:**
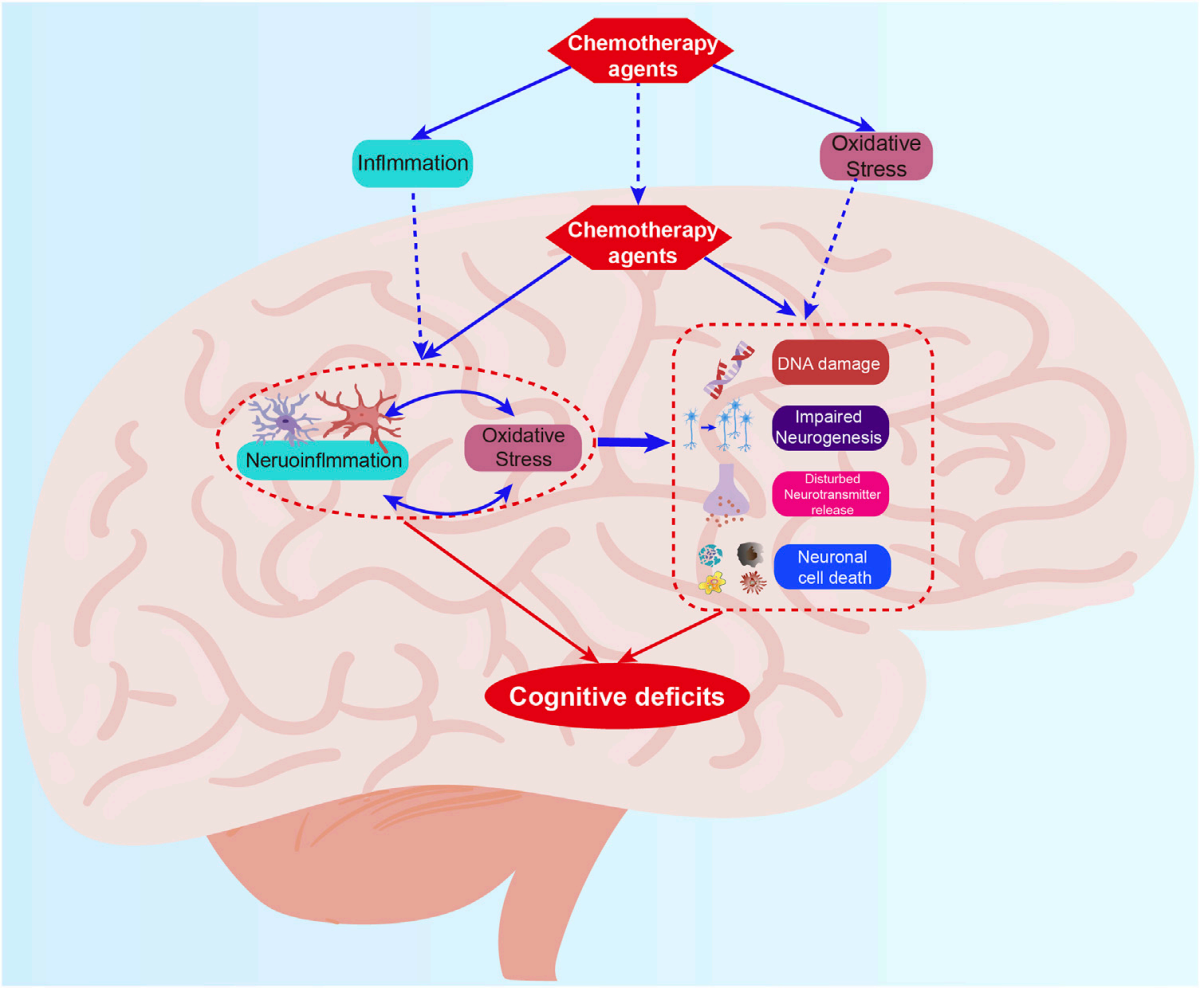
The mechanism summary of CRCI. The mechanisms mainly include oxidative stress, neuroinflammation, DNA damages, impaired neurogenesis, disturbed neurotransmitter release, and neuronal cell death which work together to induce CRCI.

The generation of reactive oxygen species (ROS) and free radicals during cellular metabolism is a fundamental process that is usually balanced by endogenous antioxidant systems (Costantini, 2019). The brain consumes a large amount of oxygen to conduct physiological processes, resulting in the elevation of free radical generation (Singh et al., 2019). Excessive free radical production leads to oxidative stress, which is responsible for oxidative injury of neurons and membranes and eventually results in cell death. Previously, oxidative stress has been defined as one of the primary mechanisms in diverse CNS diseases, including Parkinson’s disease, Alzheimer’s disease, and cerebral ischemia (Barodia et al., 2017; Poprac et al., 2017; Liu et al., 2020). The US-Food and Drug Administration (FDA) has approved 132 anti-cancer drugs, of which 56 have the potential to induce the generation of ROS (Myers et al., 2008). Therefore, oxidative stress is considered to be a cornerstone chemotherapeutic agent and is one of the pathophysiological mechanisms of CRCI (Figure 1).

In lung cancer patients, the 8-Oxo-7,8-dihydro-2′-deoxyguanosine (8-oxodG) levels in urine remarkably increase after radiotherapy and six cycles of chemotherapy, indicating that inducing the generation of free radicals underlies the anti-cancer activity of these chemotherapeutic drugs (Crohns et al., 2009). Cadeddu et al. assessed the influence of epirubicin on ROS formation in blood samples and the levels of antioxidant enzyme glutathione peroxidase in red blood cells in breast or endometrial cancer patients. The results suggested that epirubicin elevated ROS production and decreased glutathione peroxidase levels, revealing that epirubicin caused oxidative stress (Cadeddu et al., 2010). In clinical, the toxicity caused by cisplatin seems to be mainly caused by the free radical formation, resulting in oxidative organ damage (Weijl et al., 1998; Weijl et al., 2004). The cisplatin treatment can decrease the levels of antioxidants vitamins C, E, and ceruloplasmin in plasma.

Additionally, the essential role of oxidative stress has also been confirmed in experimental models. For example, treatment of DOX intraperitoneally significantly upregulates the levels of protein carbonyl and 4-hydroxy-nonrenal (4-HNE) in the brain of the mice (Keeney et al., 2018). Adriamycin administration can downregulate the levels of the antioxidant glutathione, reduce the oxidized glutathione ratio, and upregulate the expression of the pro-oxidant enzyme glutathione peroxidase (Joshi et al., 2010). The combination of DOX and CYP intraperitoneally injection decreases the glutathione and glutathione disulfide ratios in rat hippocampus tissues (Kitamura et al., 2021). The animals exposed to methotrexate also possess overproduction of lipid peroxidation in the plasma and decreased glutathione in brain regions (Rajamani et al., 2006). The increasing levels of oxidative stress in the hippocampus induced by cisplatin may lead to dendrite loss, mitochondrial DNA damage, and neuronal cell death (Lomeli et al., 2017). In rodents, CYP has been shown to be able to upregulate oxidative stress in the CNS, reflected by increased malondialdehyde levels, and decreased catalase and glutathione expression (Oboh et al., 2011; McElroy et al., 2020). In addition, CYP also inhibits the activity of catalases in the brain, heart, and lung as well as the antioxidant potential in plasma (Ince et al., 2014).

Taken together, the evidence showed that chemotherapeutic drugs could increase oxidative stress which then contributes to CRCI directly or indirectly.

### 2.2 Neuroinflammation

Normally, the immune response and the release of cytokines are controlled. It’s defined that inflammatory cytokines, such as Interleukin 6 (IL-6), Tumour necrosis factor alpha (TNF-α), and Interleukin 1β (IL-1β) are closely related to brain function, and the high levels of these cytokines can lead to changes in cognitive function (Wardill et al., 2016). In fact, increased cytokine and involved neuroinflammation have been speculated to be one of the candidate mechanisms of chemobrain.

In clinical practice, multiple studies have confirmed that chemotherapy drugs increasing cytokine production such as TNF-α, Monocyte chemoattractant protein-1 (MCP-1), Interleukin 10 (IL-10), IL-6, and Interleukin 8 (IL-8) in cancer patients and this phenomenon is more prominent in patients who experienced dyscognition (Tsavaris et al., 2002; Meyers et al., 2005; Janelsins et al., 2012). In breast cancer patients, the serum levels of TNF-α and IL-6 are significantly elevated and the hippocampal volume is reduced following chemotherapy (Kesler et al., 2013). Similarly, Zhao and colleagues also observed higher serum levels of TNF-α, IL-1β, and Interleukin 4 (IL-4) in breast cancer patients after chemotherapy. Interestingly, cancer patients with impaired cognition also possess higher levels of these cytokines. Furthermore, it has been reported that cognitive function and cytokine levels are inversely correlated in early-stage breast cancer patients with chemotherapy (Zhao et al., 2020a).

Microglia and astrocytes, the main innate-immune cells in CNS, can react to a full range of pathological stimulation. Chemotherapy agents including docetaxel (DTX) (Breedveld et al., 2006), cisplatin (Ginos et al., 1987), carmustine (Mitsuki et al., 1991), oxaliplatin (OXP) (Jacobs et al., 2010), paclitaxel (Gangloff et al., 2005), 5-FU (Sakane et al., 1999), and CYP (Seruga et al., 2008) have been reported to be able to penetrate the blood-brain barrier, which might induce hippocampal inflammation. Concordantly, experimental studies reveal that these agents enhance inflammatory cytokines production and subsequently lead to cognitive impairment. Recently, accumulating evidence indicates that chemotherapeutic drugs can promote neuroinflammation through both the peripheral and central manners. It has been found that the administration of chemotherapeutic drugs such as cisplatin, methotrexate, oxaliplatin, vincristine, and paclitaxel can increase peripheral inflammatory cytokines (Brandolini et al., 2019). Subsequently, the elevated peripheral cytokines penetrate the blood-brain-barrier (BBB) and then activate microglia and astrocytes, resulting in the promoted secretion of pro-inflammatory mediators, which hamper the neurogenesis and myelination process and eventually cause cognitive deficits. A recent study also reports that PLX5622 with specific elimination of microglia can alleviate methotrexate-related memory impairment (Geraghty et al., 2019). Collectively, chemotherapeutic drugs can directly or indirectly cause neuroinflammation and targeting microglia and astrocytes may be an attractive strategy for treating CRCI.

### 2.3 DNA damages

Cells exposed to exogenous mutagens or endogenous ROS might lead to DNA damage and to prevent these deleterious damages organisms have diverse DNA repair pathways. The failure of the DNA repairment may result in DNA mismatches, crosslinks or even DNA double-strands breaks, leading to cell death or oncogene activation. In clinical, chemotherapeutic agents, such as alkylating or antibiotic agents, kill cancer cells by evoking cell apoptosis in a damaging genomic DNA way. For example, DOX exerts its ability to kill cancer cells by cross-link with DNA to disrupt the cycle of cancer cells (Pei et al., 2020). Cisplatin can kill cancer cells though binding DNA to form adducts and subsequently induce DNA damage (Wang and Lippard, 2005). Besides, other chemotherapeutic drugs such as 5-FU, CYP, and OXP are also closely related to the induction of DNA damages (Hochster and Sargent, 2016; Park et al., 2020; Chen et al., 2021). Meanwhile, accumulated evidence correlates DNA damage with neurodegeneration and cognitive impairments (Jeppesen et al., 2011). These chemotherapy agents can induce DNA damage not only in cancer cells but also in normal and non-cancerous cells, which might be involved in the progression of CRCI.

Recently, Torre et al. first highlighted that in human frontal lobe cortical neurons, the generation of DNA damage contributed to the progression of CRCI. They found that there were higher levels of DNA damage in cortical neurons compared to cancer patients who do not receive chemotherapy or healthy (Torre et al., 2021). Furthermore, experimental studies also provided valuable insights into the involvement of DNA damage in CRCI. It has been shown that doxorubicin could disrupt the DNA double-strand structure as well as induce DNA cross-linking through accumulating in the nucleus of neurons. They found that in primary cortical neurons, doxorubicin treatment markedly reduced the expression of breast cancer type 1 susceptibility protein which is the key factor for DNA repair (Manchon et al., 2016). Moreau et al. also reported that doxorubicin remarkably elevated the levels of 8-OH(d)G which is the marker of DNA/RNA oxidation in the hippocampus of rats (Bagnall-Moreau et al., 2019). Similarly, the positively charged metabolites produced by the hydrolysis of cisplatin could bind to DNA and lead to DNA cross-linking and prevent DNA synthesis. Blocking the binding of cisplatin to DNA could effectively reduce its toxicity in both cancer cells and neurons (Fischer et al., 2008).

### 2.4 Impaired neurogenesis

The mammalian brain retains a high degree of neurogenesis in adulthood, which primarily occurs in the hippocampal dentate gyrus and the subventricular zones (Sung et al., 2020). Neurogenesis plays fundamental roles in numerous hippocampus-dependent functions, including learning, emotions, and cognition. Growing evidence documented that reduced neurogenesis was involved in the development of aging and neurodegeneration. The reduction and quiescence of neural precursor cells resulted in the decrease of neurogenesis with advancing age (Babcock et al., 2021). Clinical and experiment studies also observed that the dysbiosis of neurogenesis occurred in Alzheimer’s disease patients and animal models.

Chemotherapeutic drugs have been verified to accelerate aging and neurodegeneration, suggesting that CRCI may be closely associated with impaired neurogenesis (Seigers et al., 2008). Since conventional chemotherapeutic agents mainly function to prevent cancer cell division, they can also hamper neurogenesis. Chemotherapeutic drugs including docetaxel, vinblastine, and paclitaxel can target microtubule-related proteins, which are necessary for neuronal transport and functions (Schneiderman, 2004). Besides, it has been reported that chemotherapeutic drugs, including doxorubicin, cyclophosphamide, carmustine, cisplatin, and 5-fluorouracil can disrupt hippocampal neurogenesis by altering various protein markers. For example, a single dose of methotrexate injection (37.5–300 mg/kg) obviously reduces Ki-67-positive cells in the hippocampus (Seigers et al., 2008). Nokia et al. have found that temozolomide exposure downregulated the bromodeoxyuridine (BrdU)-positive cell numbers (Nokia et al., 2012). Interestingly, 5-fluorouracil treatment shows little effect on the Ki-67-positive cells but remarkably inhibits doublecortin (DCX)-positive cell number, indicating that early neuronal neogenesis is affected (Mustafa et al., 2008). Besides, cyclophosphamide or doxorubicin administration can inhibit the expression of both DCX- and BrdU-positive cells, indicating that both neuronal neogenesis and maturation were influenced (Christie et al., 2012). Therefore, different chemotherapeutics may affect neurogenesis in different manners. Considering that brain-derived neurotrophic factor (BDNF), a kind of neurotrophin, is a crucial mediator for regulating synaptic plasticity, neuronal survival, differentiation, and neurogenesis. Although the mechanisms of BDNF loss-induced neurogenesis dysfunction are still unclear, it has been reported that low levels of BDNF in the serum were associated with cognitive impairment in patients with cancer and animal models (Jehn et al., 2015; Zimmer et al., 2015). Moreover, the therapeutic strategies to resume the BDNF levels in the brain have also been described and provide their scope of implication to improve neurogenesis and treat CRCI.

### 2.5 Disturbed neurotransmitter release

The dysregulation of neurotransmitters is a common feature of most neurological disorders. For example, a decrease of acetylcholine (Ach) is often detected in patients with AD. During aging, dopaminergic neurons are reported to decline by about 5%–10% per decade. Notably, the most common neurological drugs improve cognitive deficits by modulating neurotransmitter release. Many evidence has shown that chemotherapy can reduce neurotransmitter production and release in brain tissues (Fitzgerald, 2021; Rao et al., 2022). As we know, Ach, a key cholinergic neurotransmitter, can sustain brain function by regulating long-term potentiation. During the synthesis of Ach, phospholipase D (PLD) firstly catalyzes phosphatidylcholine (PtdCho) to release choline which is subsequently acetylated by choline acetyltransferase (ChAT), and ultimately, Ach is formed (Ferreira-Vieira et al., 2016). It has been reported that the mice exposed to DOX possess reduced Ach production, inhibited PLD and ChAT, as well as decreased choline-containing compounds (Lim et al., 2016; Keeney et al., 2018). Moreover, DOX-induced oxidative stress and TNF-α expression can also increase acetylcholinesterase (AChE) activity and inhibit PtdCho synthesis (Keeney et al., 2018). Cisplatin can also increase AChE activity in neural tissue homogenates (Song et al., 2010). Interestingly, it has also been reported that CYP or cisplatin are related to reduced AChE activity in the hippocampus (Oz et al., 2015; Lim et al., 2016). Nicotinic signaling has also been implicated in CRCI. It is reported that CYP and DOX co-exposure can reduce α7 nicotinic acetylcholine receptor (nAChR) mRNA expression in the hippocampus (Kitamura et al., 2017). Continine, the main derivative of nicotine which is known as an α7nAchR ligand, can reverse cognitive and depressive behaviors in CRCI models (Iarkov et al., 2016). Besides, chemotherapeutic agents can also alter glutamate levels in the brain. DOX administration impedes glutamate clearance reflected by the decreased rate of glutamate uptake in the frontal cortex (Thomas et al., 2017). It’s considered that the declined glutamate clearance may be contributed by the downregulated-glial transport proteins or excessive glutamate production in astrocytes (Thomas et al., 2017). In the synapse, the increased glutamate will bind with N-methyl-d-aspartate (NMDA) rceptors, resulting in elevated calcium-dependent excitability and suppressed BDNF content, which ultimately promote neuronal apoptosis. It has been confirmed that 5-HTergic neurons can regulate hippocampal synaptic plasticity via 5-HT1A receptor-mediated inhibitory control. And the depletion of 5-HT impedes hippocampus-dependent declarative memory and induces poor performance in a new object recognition task (Fernandez et al., 2017). Doxorubicin has been found to be able to decrease monoamines production, serotonin (5-HT) and dopamine (DA), which are closely associated with cognitive function (Kwatra et al., 2016). MTX treatment also can decrease norepinephrine, dopamine, 5-HT, and 5-HT metabolite levels in the hippocampus (Madhyastha et al., 2002). 5-FU has been found to decrease striatal DA levels in rats (Jarmolowicz et al., 2019). Similarly, carboplatin, an alkylating chemotherapeutic agent, impairs DA reuptake and 5-HT release (Kaplan et al., 2016). Recently, several clinical studies have correlated variants of catechol-O-methyltransferase (COMT) with the disturbed neurotransmitters release during the developing chemobrain. For example, COMT can regulate the metabolism of dopamine, norepinephrine, and epinephrine (Sheldrick et al., 2008). Particularly, COMT 158Val allele is associated with elevated COMT enzymatic activity, and thereby decreased-cortical dopamine (Small et al., 2011). Consequently, the survivors carrying one or more Val alleles are more likely to develop chemobrain, which may be caused by their smaller dopamine reservoir (Small et al., 2011). Similarly, another COMT variant, rs165599 G/G, is also associated higher risk of chemobrain in breast cancer patients (Cheng et al., 2016). Thus, the disturbed neurotransmitter release is probably one of the mechanisms underlying chemobrain.

### 2.6 Neuronal cell death

In physiological conditions, the death of neuronal cells is under strict control even in older individuals. However, a significantly increased neuronal loss has been observed in many CNS diseases, which also correlates with cognitive deficits. There is extensive data supporting the role of cell death, including necroptosis, apoptosis, ferroptosis, pyroptosis, and cell death associated with autophagy, in the pathogenesis of CNS diseases. When cellular stress occurs, various types of cell death will be activated (Moujalled et al., 2021). As we know, cell death is closely associated with tumor treatment as chemotherapy is specifically designed to induce the death of cancer cells, but this is at the cost of causing the death of many healthy cells, particularly nerve cells. For example, apoptosis is known as a kind of programmed cell death. Carmustine, cisplatin, or oxaliplatin-related CRCI has been found to be related to the upregulated pro-apoptotic protein cysteinyl aspartate specific proteinase 3 (caspase-3) and caspase-9, and the downregulated antiapoptotic protein, B-cell lymphoma-2 (Bcl-2) in the brain tissues (Helal et al., 2009; Bianchi et al., 2017; Lomeli et al., 2017). DOX can also promote the interaction between Fas and Fas ligand, which subsequently recruit the Fas-associated protein death domain (FADD), culminating in the activation of apoptotic pathways (Du et al., 2021). Necrosis as an alternative cell death is charcteristiced by cell swelling. DOX has been reported to trigger necroptosis occurrence by promoting the phosphorylation of receptor-interacting protein kinase 1 (RIPK1), receptor-interacting protein kinase 3 (RIPK3), and Mixed lineage kinase domain-like protein (MLKL). Aberrant autophagy is extensively found in neurological diseases. DTX has been found to induce autophagy in the hippocampus (Fardell et al., 2014). DOX administration may impair the autophagy-lysosome system in neurons of mice (Moruno-Manchon et al., 2016). Cisplatin also can initiate autophagy via regulating the endoplasmic reticulum (ER) stress-mediated activation of the activating transcription factor 4 (ATF4)-protein kinase B (Akt)-mechanistic target of rapamycin (mTOR) signaling pathway, resulting in the emerge of apoptosis (Yi et al., 2020).

## 3 Natural products based nose to brain drug delivery (NBDD)

The blood-brain barrier is a selective and dynamic permeability barrier between the circulatory system and the brain. This border protected the brain neurons against both endogenous and exogenous toxic substances. However, it is also a significant hindrance to the transportation of medications from circulation into the CNS. Currently, there are few therapeutics effectively used in treating CNS diseases, in addition, the efficacy of the current drug is restricted for insufficient drug transportation via the BBB (Fung et al., 2016). Drug transit via BBB is largely determined by drug characteristics, such as molecular size, dissociation degree, and hydrophilicity (Bharadwaj et al., 2016). Therefore, developing novel drug delivery systems which can efficiently transport therapeutics into the CNS is in great demand for treating CNS diseases (Xie et al., 2019).

In 1989, William H. Frey II first introduced the concept of intranasal administration, which could deliver drugs directly into the CNS (Reger et al., 2006). Since then, numerous studies have been conducted to verify the feasibility of nose-to-brain drug delivery systems in animal models and clinical trials. Nasal administration is a non-invasive manner of administration with many benefits, including ease of administration, fast onset, high compliance, and voidance of the hepatic first-pass effect. Importantly, the drugs could bypass the BBB and achieve brain targeting. Over the last decades, all these benefits have established a trend toward the development of nasal-administered formulations for CNS diseases. NBDD is based on the olfactory nerve pathway, the trigeminal nerve pathway, and the blood circulation pathway, hence, the combination of these pathways is essential for nose-to-brain drug delivery.

These years, it has been defined that nanoparticles (NPs) are promising drug delivery systems to promote drugs across BBB. A full series of nanomaterials, including liposomes, micelles, nanofibers, polymeric nanoparticles, and inorganic nanoparticles have been investigated to target the brain. The use of nanotechnology in NBDD is promising. Currently, it raises our attention that nanotechnology can promote the delivery of natural compounds for treating central nervous system diseases. It has been confirmed that nanotechnology can promote drug residence at the site of absorption, as well as increase drug solubility and mucosal permeation. In addition, the systemic side effects of drugs can be circumvented by reducing drug distribution to the non-targeted area (Schneider-Futschik and Reyes-Ortega, 2021). These superior features largely promote the application of NPs for NBDD (Sonvico et al., 2018). Therefore, it’s promising to develop NPs via intranasal administration to target neural tissue for the treatment of CRCI.

## 4 Natural products for the treatment of CRCI

Studies have revealed that natural products may be beneficial for treating CRCI, particularly those compounds classified as phenols, flavonoids, terpenoids, and others (Table 1).

**TABLE 1:** The role of natural products in the treatment of Chemobrain.

Categories	Monomers	Chemical structures	Chemotherapeutic agents	Effects	Molecular mechanisms	Ref. (PMID)
Phenols	Resveratrol	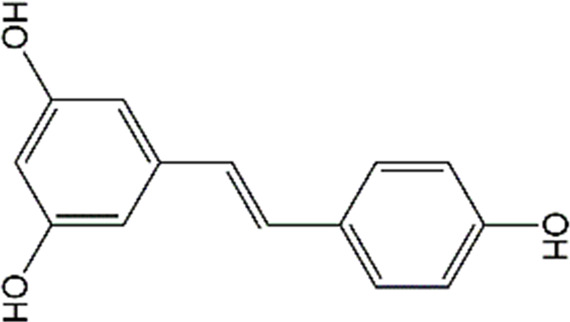	Doxorubicin	GFAP↓, IBA-1↓	Anti-inflammation	34536813
			Docetaxel, adriamycin, and cyclophosphamide	IL-6↓,TNF-α↓,IL-4↑,IL-10↑ GABA_A_R↑, NMDAR1↑, p-CaMKII↓, BDNF↑,TrkB↑	Anti-inflammation; Improve neuroplasticity	29534932
	Polydatin	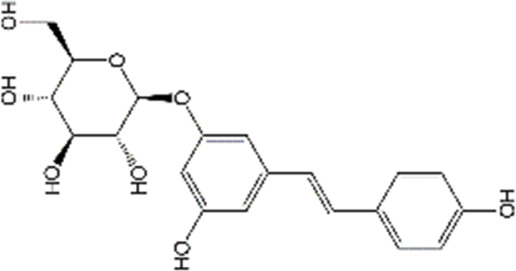	Doxorubicin	MDA↓, GSH↑, TNF-α↓, PGE-2↓, COX-2↓, cleaved caspase-3↓, cleaved caspase-9↓	Anti-inflammation; Anti-apoptosis; Anti-oxidative stress	31992173
	Epigallocatechin-3-gallate	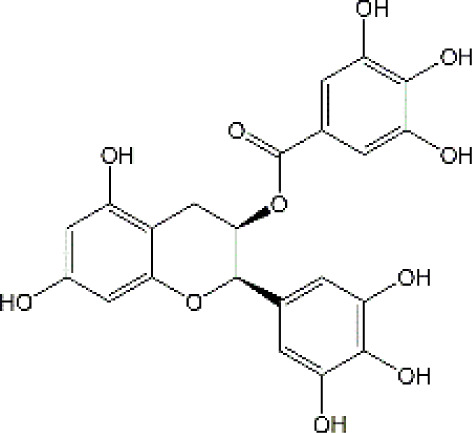	Cisplatin	IL-6↓, TNF-α↓, iNOS↓, MDA↓, NO↓, TAC↑, Cleaved caspase-3↓,Bax↓, Bcl-2↑, BDNF↑, AChE↑, ACh↓	Anti-inflammation; Anti-apoptosis; Anti-oxidative stress; Improve neurotransmitter release	31410684
	Curcumin	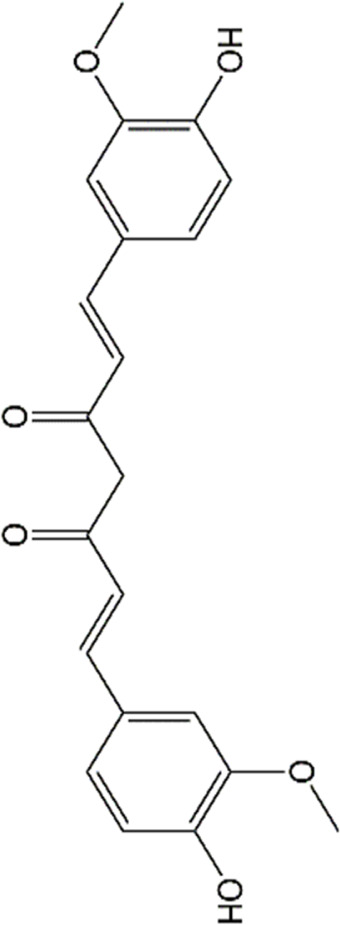	Cisplatin	Bax↓, Bcl-2↑, Bim↓, LC3-II/LC3-I↑	Improve neurogenesis and synaptogenesis Increase autophagy	31843707
			Cisplatin	MDA↓,SOD↑, AChE↑	Anti-oxidative stress	25982942
			Doxorubicin	GFAP↓, IBA-1↓	Anti-inflammation	34536813
	nanocurcumin		Doxorubicin	MDA↓,GSH↑, NO↓, AChE↑,MAO↑	Anti-oxidative stress	33882267
			Cisplatin	MDA↓,GSH↑, NO↓, caspase-3↓, TNF-α↓, AChE↓	Anti-inflammation; Anti-apoptosis; Anti-oxidative stress; Improve neurotransmitter release	30257586
	Caffeic acid phenethyl ester	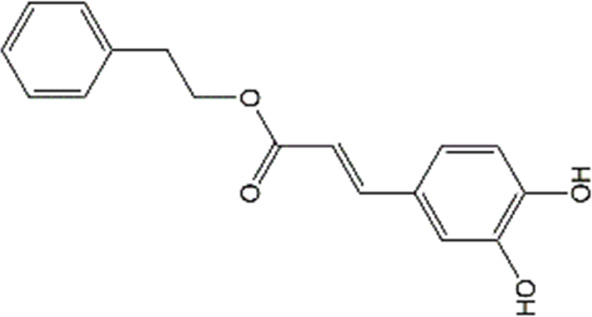	Doxorubicin	MDA↓,GSH↑, GFAP↓, COX-2↓, TNF-α↓,ACh↑, caspase-3↓,	Anti-inflammation; Anti-apoptosis; Anti-oxidative stress	33011199
	Sulforaphane	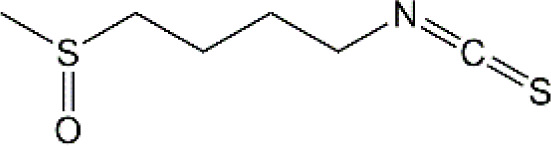	Cisplatin	AChE↓, LPO↓, GSH↑,NO↑	Anti-oxidative stress; Improve neurotransmitter release	35969308
Flavonoid	Luteolin	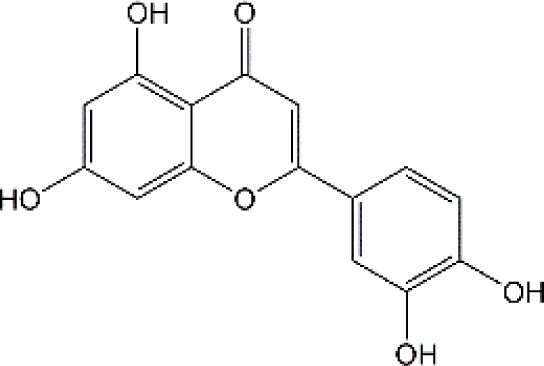	Doxorubicin	CAT↑, SOD↑,GST↑,GPX↑,GSH↑,TSH↑,LPO↓,RONS↓,XO↓, AChE↓,NO↓,MPO↓, TNF-α↓,IL-1β, IL-10↑, caspase-3↓	Anti-inflammation; Anti-apoptosis; Anti-oxidative stress	34766659
	Quercetin	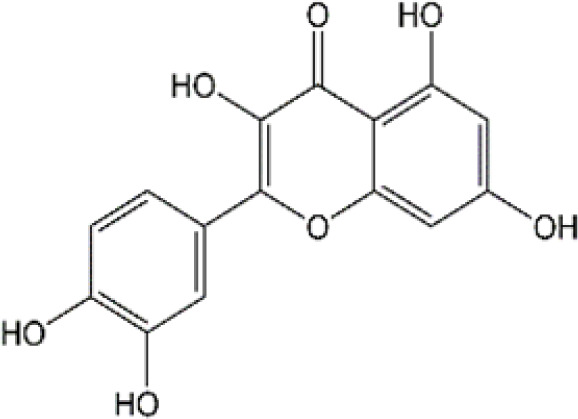	Adriamycin	corticosterone↓, GST↑, GSH↑, MDA↓, lymphocytes↑, leukocytes↑	Anti-oxidative stress; Improve immune dysfunction	24947870
			Cyclophosphamide and doxorubicin	SOD↓, CAT↑, GSH↑, MDA↓	Anti-oxidative stress	25260542
	Naringin	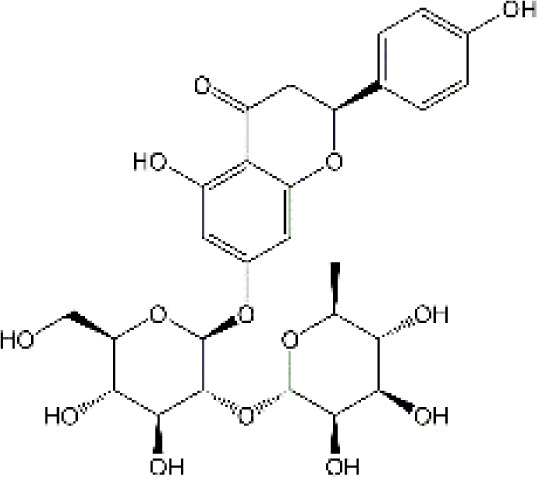	Cisplatin	AChE↓, MDA↓, PCO↓, H2O2↓,ROS↓, nitrite formation↓, iNOS↓, GSH↓, Ascorbic acid↓,SOD↑,CAT↑,GPx↑	Anti-oxidative stress; Anti-inflammation; Improve neuroplasticity	25896911
			Doxorubicin	MDA↓, SOD↑, GSH↓, CAT↑, IL-1β↓,TNF-α↓	Anti-oxidative stress; Anti-inflammation	27209303
	Ellagic acid	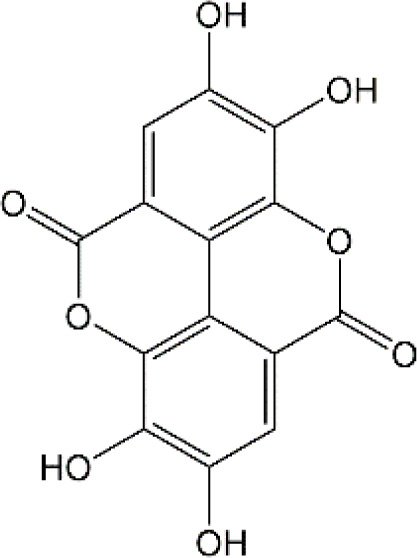	Doxorubicin	MDA↓, GSH↓, TNF-α↓, iNOS↓, CHE activity↓, 5-HT↑, DA↑, NE↑, caspase-3↓	Anti-oxidative stress; Anti-inflammation; Improve neurotransmitter release	28815802
	Rutin	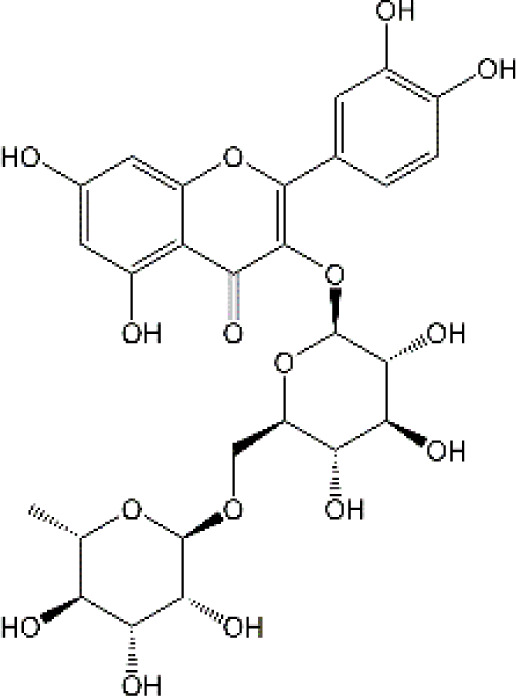	Doxorubicin	Catalase↑, SOD↑, GSH↑, Total thiols↑, TNF-α↓, ROS↓, Apoptosis↓, neurite length↑, neurite width↓, cell viability↑	Anti-oxidative stress; Anti-inflammation; Anti-apoptosis; Improve neuroplasticity	28408800
			Doxorubicin		Anti-oxidative stress; Anti-inflammation	31679278
			Cisplatin	TBARS↓, glutathione↑, GPX↑	Anti-oxidative stress	28962559
	Morin	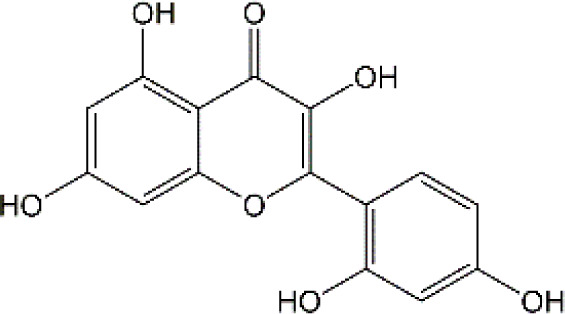	Doxorubicin	MDA↓, GSH↑, SOD↑, CAT↑, GPx↑, TNF-α↓, IL-1β↓,Bcl-2↑, Caspase-3↓, AChE↓, GFAP↓	Anti-oxidative stress; Anti-inflammation; Anti-apoptosis	29990832
	Epicatechin	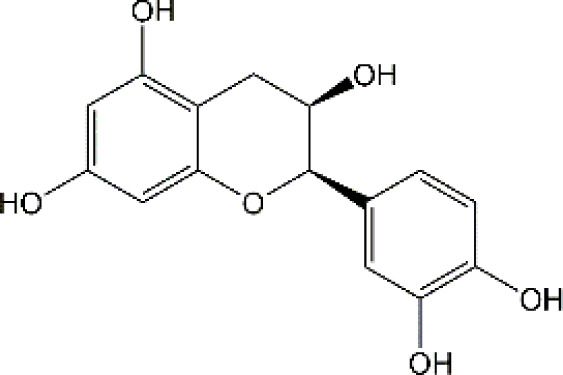	Doxorubicin	CAT↑, SOD↑, GSH↑, MDA↓, Catalase↑, GFAP↓, TNF-α↓, iNOS↓	Anti-oxidative stress; Anti-inflammation	21763406
	Juglanin	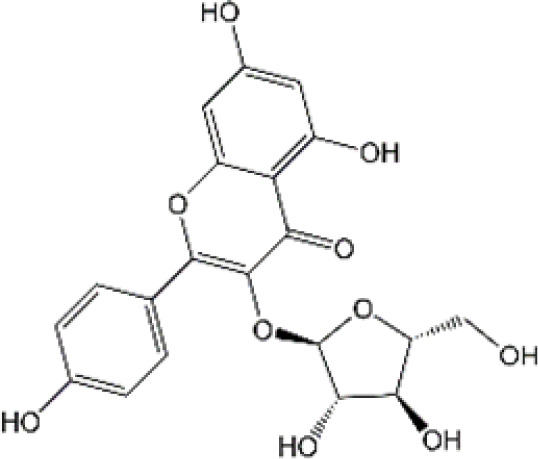	Doxorubicin	AchE↑, SOD↑, GSH↑, CAT↑, MDA↓, IL-6↓, IL-1β↓, TNF-α↓, Caspase-3↓	Anti-inflammation; Anti-apoptosis; Anti-oxidative stress; Improve neuroplasticity	35138546
	Galangin	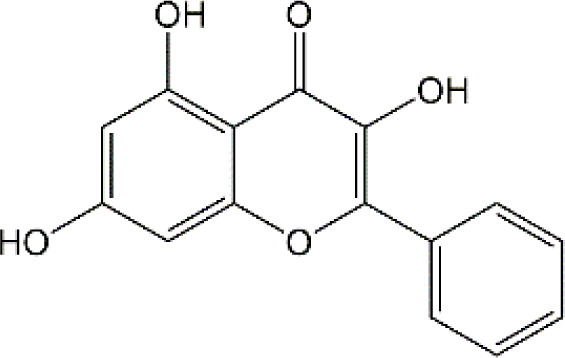	Doxorubicin	MDA↓,NO↓, GSH↑, TNF-α↓, IL-1β↓, IL-6↓, iNOS↓, HMGB1↓, GFAP↓, BDNF↑	Anti-inflammation; Anti-necroptosis; Anti-oxidative stress; Improve neurotransmitter release	35843304
Terpenoids	Ginsenoside	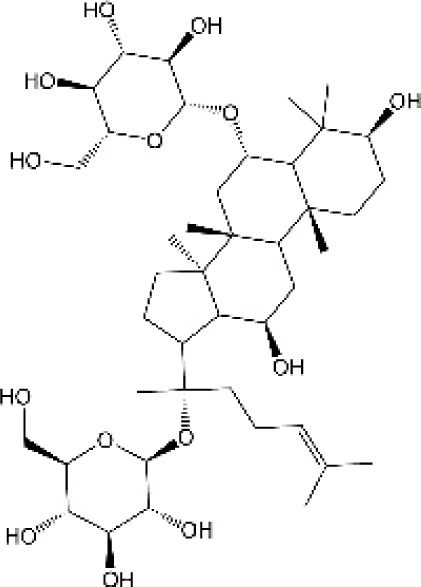	Docetaxel, adriamycin, and cyclophosphamide	TNF-α↓, IL-6↓, IL-10↑, IL-4↑, GFAP↓,IBA-1↓, BDNF↑	Anti-inflammation; Improve neuroplasticity	30659419
			Cisplatin	SOD↑, GSH↑, MDA↓,ROS↓, TNF-α↓, IL-10↑, IL-1β↓, AchE↓, Ach↑,chAt↑	Anti-inflammation; Anti-oxidative stress; Improve neuroplasticity	31695559
	Asiatic acid	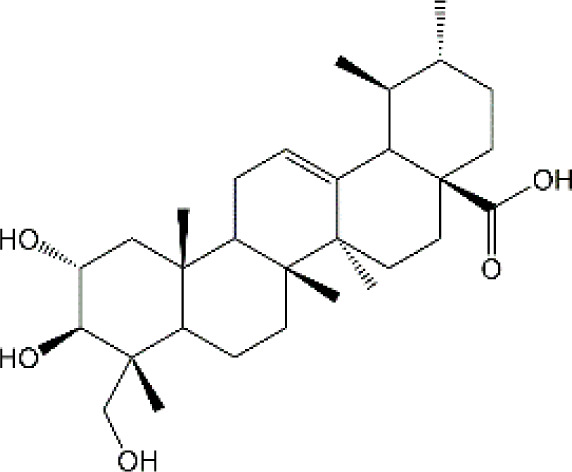	5-fluorouracil	Ki-67 positive cells↑, BrdU positive cells	Improve neurogenesis	28700628
			5-Fluorouracil	p21 positive cells↑,MDA↓	Anti-oxidative stress; Improve neurogenesis	
	Ganoderic acid	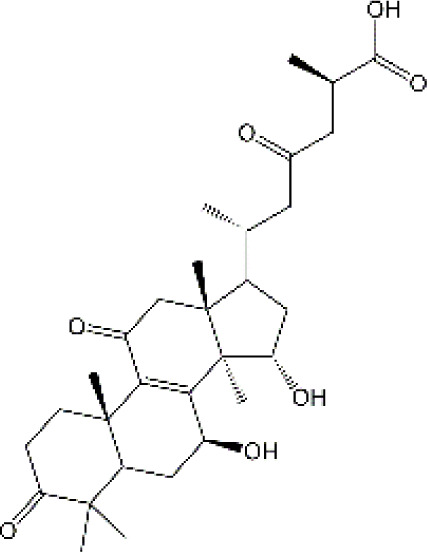	5-fluorouracil	IL-6↓, IL-1β↓, iNOS↓, COX2↓, BDNF↑	Anti-inflammation; Improve neuroplasticity	34821902
	Astaxanthin	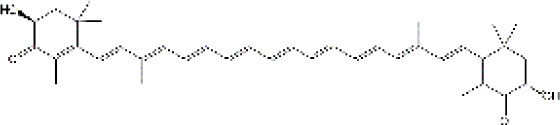	Doxorubicin	AChE↓, TNF-α↓,PGE2↓,COX-2↓,GFAP↓, Caspase-3↓,cytochrome c↓	Anti-inflammation; Anti-apoptosis	29039023
Others	CI protein	NA	Doxorubicin	AchE↓,ROS↓,MAO↓,NE↑,DA↑,5-HT↑, MDA↓, SOD↑, GST↑, GR↑, GPx↑, GSH↑, GSSG↓, cleaved caspase-3↓, Cleaved PARP↓, Bad/Bcl-2↓, cytochrome↓, caspase 9↓, Apaf1↓	Anti-inflammation; Anti-necroptosis; Anti-oxidative stress; Improve neuroplasticity	22448708
	C-phycocyanin	NA	Doxorubicin	TNF-α↓, IL-1β↓, IL-6↓, GFAP↓, IBA1↓, MDA↓, Protein carbonyl↓, 8-OHdG↓, GSH↑, SOD↑, Sypnapsin-1↑, PSD95↑	Anti-inflammation; Anti-oxidative stress; Improve neuroplasticity	33237471
	Astragali Radix	NA	Doxorubicin	MDA↓, SOD/MDA↑, Improve amino acid homeostasis	Anti-oxidative stress; Improve amino acid homeostasis	33945017
	Extract of Tiliacora triandra	NA	Cisplatin	MDA↓, GPx↑, SOD↑, GSH↑, CAT↑, AchE↓, TNF-α↓, IL-6↓, IL-1β↓, Caspase-3↓, Bcl-2↑, p53↓	Anti-inflammation; Anti-necroptosis; Anti-oxidative stress; Improve neuroplasticity	34916822
	Ergothioneine	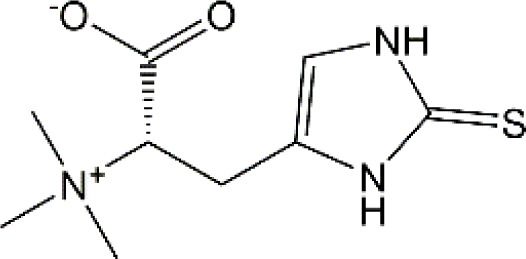	Cisplatin	AchE↓, MDA↓, GSH/GSSG ratio↑	Anti-oxidative stress; Improve neuroplasticity	20932872
	Bixin	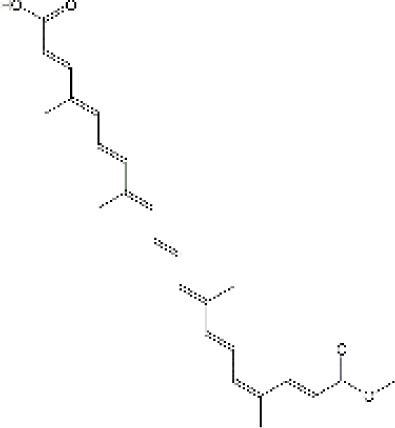	Cisplatin	Cell viability↑, micronucleus frequency↓, DNA in tail↓	Anti-DNA damage	22019694

### 4.1 Phenols

#### 4.1.1 Resveratrol

Resveratrol (RSV), a natural polyphenolic substance, is one of the primary ingredients in the herb Polygonum cuspidatum Sieb.et Zucc. Currently, RSV is one of the phytochemicals that has been widely investigated and has various biological activities, such as anti-apoptotic, antioxidant, anti-inflammatory, and, anticancer properties (Miguel et al., 2021). In terms of brain-related ailments, both experimental and clinical investigations have shown that RSV has beneficial effects on neurodegenerative diseases, ischemic stroke, and depression (Gomes et al., 2018; Gu et al., 2019; Khoury et al., 2019). In rats intraperitoneally injected with DOX (2.5 mg/kg/week) for 4 weeks, RSV (10 mg/kg/day) effectively improved the short- and long-term memory impairments as well as alleviated microglial and astrocyte over-activation in the cortex, hypothalamus, and hippocampus (Moretti et al., 2021). For breast cancer, docetaxel, adriamycin, and cyclophosphamide (DAC) are commonly used in combination. Shi and colleagues showed that pretreatment with RSV (50 and 100 mg/kg/day) for 1 week significantly decreased pro-inflammatory cytokines expression, increased neuronal activities in pre-front and hippocampus, elevated the neuroplasticity biomarkers expression, and improved cognitive impairment in mice treated with DAC (Shi et al., 2018). However, it is arduous to translate RSV into clinical utilities owning to the low stability, high metabolism, and poor bioavailability (Amri et al., 2012). Furthermore, although RSV has been shown to possess the ability to translate from blood to brain in rodents and humans (Wang et al., 2002; Turner et al., 2015), the distribution of RSV in the central CNS is very poor for the existence of the blood-brain barrier and choroid plexus (Ganesan et al., 2015). Interestingly, a recent study has shown that the administration of RSV via the nose can reach cerebrospinal fluid through the olfactory region (Pandareesh et al., 2015). Furthermore, RSV has a high membrane permeability based on its high oil-water partition coefficient, therefore, RSV is suitable for designing as NBDD preparation (Hao et al., 2016). For example, Trotta et al. (2018) found that nasal administration of the chitosan-coated lipid microparticles with resveratrol could markedly increase the bioavailability of RSV in the cerebrospinal fluid and decrease the RSV distribution in the bloodstream, indicating that the NBDD can directly delivery RSV to the brain. Salem and colleagues also developed an RSV-loaded intranasal transpersonal mucoadhesive gel, which significantly enhanced RSV bioavailability and achieve direct nose-to-brain targeting (Salem et al., 2019). Collectively, the above results suggested that RSV is a promising agent for the treatment of CRCI and the administration of RSV via NBDD would promote its efficiency via the increased bioavailability and alleviated adverse effects.

#### 4.1.2 Polydatin

Polydatin, also known as piceid (3,4′,5-trihydroxystilbene3-β-D-glucoside), is a natural stilbene that primarily existed in Polygonum cuspidatum Sieb. et Zucc. Numerous preclinical studies have reported that polydatin possesses anti-inflammatory and antioxidant abilities (Tang et al., 2022). Polydatin can scavenge free radicals and then protect cardiomyocytes through regulating lipid metabolism, and antagonizing platelet aggregation (Wu M. et al., 2020). Furthermore, due to its anti-inflammatory and anti-oxidant effects, polydatin exerted protective activities against ischemia-reperfusion injury in many organs, such as the heart, kidneys, lungs, and cerebellum (Sun and Wang, 2020). In terms of chemobrain, previous studies have pointed out that polydatin (50 mg/kg/day) can mitigate DOX-induced learning and memory deficits, and protect the hippocampal architecture from damage. Further mechanistic studies reveal that polydatin administration in DOX-treated rats restores the nuclear factor-erythroid 2-related factor 2 (Nrf2) levels and reduces the nuclear factor kappa-B (NF-κB) expression in the hippocampus. Overall, polydatin can alleviate cognitive impairment and improve neurological function via its anti-inflammatory and antioxidant activities. Although earlier studies have reported that polydatin and its metabolites can cross the BBB, the potential drawbacks of pharmacokinetic, including low selectivity, rapid metabolism, and poor bioavailability limit its clinical application (Huang et al., 2018; Fakhri et al., 2021). Recently, it has been reported that polymeric nanocapsules loaded with polydatin exhibit comparable inhibitory effects on inflammatory responses and oxidative stress in lipopolysaccharide (LPS)-treated neurons as naïve polydatin with preserving the free form of polydatin (Basta-Kaim et al., 2019). Other novel delivery systems of polydatin, for example, polydatin-loaded liposomes could enhance the release profile of polydatin, which markedly improve the polydatin bioavailability and extend drug circulation time (Wang X. et al., 2015). However, there were no studies focusing on polydatin-related NBDD in neuroprotection. Therefore, more researches are needed to explore the protective effects of polydatin on chemobrain, especially through NBDD.

#### 4.1.3 Epigallocatechin-3-gallate

Epigallocatechin-3-gallate (EGCG), a major active polyphenol in green tea, has been demonstrated to possess neuroprotective effects in neurodegenerative diseases and neural injury. These pharmacological activities are mainly ascribed to their antioxidative, anti-inflammatory, and antiapoptotic properties. As we know, EGCG is one of the most effective phenolic compounds in free radical scavenging. According to statistics, EGCG remarkably decreases the levels of nitrosative and oxidative stress in the brain cortex after cisplatin exposure, through suppressing the contents of Malondialdehyde (MDA) as well as elevating the nitric oxide (NO) and total antioxidant capacity (TAC) levels (Arafa and Atteia, 2020). On the other hand, treatment with EGCG significantly decreased pro-inflammatory cytokines (TNF-α and IL-6) levels in the brain cortex tissue. Meanwhile, EGCG also exerted anti-apoptotic effects with increasing Bcl-2 expression and suppressing Bcl-2-associated X (Bax) and caspase-3 expression in rat exposured to cisplatin (Arafa and Atteia, 2020). BDNF, as a member of neurotrophins, play a significant part in regulating synaptic plasticity, survival, differentiation, and regeneration. Interestingly, both BDNF and AChE showed positive correlations with TAC and Bcl-2 in the brain cortex. In contrast, remarkable negative correlations were observed between the expression of BDNF, and AChE and the production of NO, MDA, TNF-α, IL-6, inducible nitric oxide synthase (iNOS), Bax, and caspase-3. To sum up, EGCG may exert protective roles in cisplatin triggered-chemobrain. Despite the fact that EGCG is beneficial in CRCI, EGCG application is limited because of the poor pharmacokinetics which is reflected by low bioavailability, fast metabolism, and rapid removal (Alam et al., 2022). To improve EGCG pharmacokinetics, a series of EGCG nanoformulations are currently emerging due to the colloidal stability, advanced tissue permeability, and drug bioavailability (Mehmood et al., 2022). For instance, chitosan-based EGCG significantly promoted the uptake and retention time of intestinal epithelium and improved the effectiveness of EGCG against atherosclerosis (Hong et al., 2014). Transdermal EGCG gel also significantly increased plasma levels and prolonged its half-life (Lambert et al., 2006). In terms of neuroprotection, zheng et al. demonstrated that EGCG-loaded liposomes (phosphatidylserine-EGCG-liposomes and phosphatidylserine-EGCG-Vitamin E-liposomes) significantly alleviated LPS-induced neuroinflammation *in vitro* and Parkinson rat models (Cheng et al., 2021). Other drug delivery methods on NPs-based EGCG include intravenous, ocular, and intratumoral routes. However, there is no report about EGCG for the treatment of CNS disorders via NBDD. Further study on the NBDD application in enhancing EGCG credibility in treating CRCI is needed.

#### 4.1.4 Curcumin

Curcumin (CUR), the principal polyphenol of the traditional medicine known as turmeric, is one of the widely studied phytochemicals with a wide range of health benefits. Experimental studies have reported that CUR exhibits neuroprotective effects in a series of CNS diseases, including traumatic brain injury, cerebral ischemic damage, neurodegenerative diseases, and cognitive deficits caused by toxicants (Bhat et al., 2019; Khayatan et al., 2022). Epidemiological research also confirmed that the long-term use of CUR is safe and ameliorates cognitive dysfunction in the elderly (Ng et al., 2006; Cox et al., 2015). It has been shown that CUR protected the brain from damage principally by attenuating microglia/astrocyte activation-mediated neuroinflammatory responses, inhibiting free radical production, alleviating blood-brain barrier disruption, and improving cerebral blood flow et al. (Reddy et al., 2018; Fan and Lei, 2022). Regarding the protective effects of CUR on chemobrain, recent research by Yi and colleagues showed that CUR could protect against cisplatin-triggered neurotoxicity in mice (Yi et al., 2020). In the study, administration of CUR (100 mg/kg) effectively alleviated cisplatin-induced cognitive deficits by regulating apoptosis-related proteins, promoting autophagy, and initiating neurogenesis and synaptogenesis. Oz et al. also reported that the rats exposed to cisplatin (5 mg/kg/week, 5 weeks) resulted in cognitive deficits, increased MDA contents, and inhibited superoxide dismutase (SOD) activities in the hippocampus. However, supplementation of CUR (300 mg/kg/day, 5 weeks) improved cognition, decreased oxidative stress, and restored cholinergic functions (Oz et al., 2015). Recently, several studies also demonstrated that CUR effectively antagonized the neurotoxicity induced by DOX. For example, Moretti and coworkers found that DOX exposure (2.5 mg/kg/week, 4 weeks) impaired short- and long-term memory in rats, while CUR via oral administration (100 mg/kg/day) for 28 days significantly reversed DOX-triggered astrogliosis, microgliosis, and memory loss (Moretti et al., 2021). Taken together, CUR possesses certain anti-chemobrain effects, antioxidant, and anti-inflammatory effects. Nevertheless, CUR application is restricted by the fact of its insufficient bioavailability and rapid metabolism. Therefore, it is essential to enhance the effective concentration of CUR in the brain to ensure its successful application. The nanotransformation of CUR is considered a strategy to improve its efficacy in the brain (Yallapu et al., 2012; Chen et al., 2013). It has been reported that CUR could cross the BBB to enter brain tissue, however, CUR nanoparticulation possessed more retention time in the hippocampus and cerebral cortex than CUR (Tsai et al., 2011). Besides, the CUR nanoparticulation has a 10–14 fold higher absorption rate than free CUR (Yallapu et al., 2012). The nanoparticle formulation of CUR significantly increased its absorption rate, bioavailability, and plasma concentration (Cheng et al., 2013). A previous study has reported that a nanoparticle loaded with CUR effectively inhibited cisplatin (12 mg/kg)-induced inflammatory cytokines production, lipid peroxidation, and acetylcholinesterase activity (Khadrawy et al., 2019). Meanwhile, the benefits of CUR nanoparticulation in suppressing DOX-caused neurotoxicity in rat’s brain have also been reported (Khadrawy et al., 2021). More importantly, it has been revealed that the intranasal administration of CUR can block liver metabolism, which provides more benefits than the CUR administration intraperitoneally or orally (Subhashini et al., 2013). Therefore, NBDD may be a new strategy to deliver CUR to the brain. Shinde and coworkers reported that the intranasal administration of CUR microemulsion markedly has higher Cmax and area-under-the-curve plasma/serum concentrations (AUC) in the brain (Shinde and Devarajan, 2017). Chen et al. 2013 designed a kind of CUR hydrogel, and its intravenous administration remarkably promoted brain-uptake efficiency, and elevated curcumin distribution in the cerebellum and hippocampus. Madane and Mahajan reported that there was higher CUR concentration in the brain after intranasal administration of CUR-loaded nanostructured lipid carrier (Madane and Mahajan, 2016). Sintov et al. also designed amyloLipid nanovesicles loaded with CUR, which significantly increased the concentration of CUR both in the brain and plasma, alleviated amyloid-β-protein (Aβ) accumulation, and suppressed Aβ-caused inflammatory responses (Sintov, 2020). At present, the studies provide application prospects for CUR administration via NBDD as a promising strategy for treating chemobrain.

#### 4.1.5 Caffeic acid phenethyl ester

Caffeic acid phenethyl ester (CAPE), a bioactive polyphenolic compound, which is first discovered from propolis. The high permeability of CAPE allows it to be cleaved by intracellular esterases and then to release caffeic acid. As a polyphenol, CAPE contains hydroxyls within the catechol ring, which guarantees its antioxidant activities. In addition, several studies also suggested that CAPE is a potential inhibitor of NF-κB activation (Natarajan et al., 1996). All these features provide the molecular basis for its neuroprotective activities in the central and peripheral nervous systems (Cetin and Deveci, 2019). Experiment studies have shown that CAPE can markedly improve DOX-damaged learning and memory functions in passive avoidance tests and morris water maze. Furthermore, CAPE effectively reversed DOX-caused oxidative stress, which was reflected by the upregulation of GSH levels and reduced lipid peroxidation in hippocampal and cortical tissues (Ali et al., 2020). Additionally, the inhibition of astrocytes activation, pro-inflammatory cytokines (COX-2 and TNF-α) production, and NF-kB nuclear translocation by CAPE was also reported (Ali et al., 2020). Besides, it has also been revealed that CAPE markedly hampers DOX-triggered caspase-3 activation. The above research suggests that the protection of CAPE against chemobrain is attributed to its regulation of oxidation damages and neuroinflammation, which indicates that CAPE as a promising therapeutic option for chemobrain. However, although CAPE could cross the BBB, the water insolubility, rapid clearance, and short half-life restrict its clinical application. To our understanding, few studies have reported the CAPE-loaded delivery system targeting the CNS. For instance, it has been reported that the intravenous administration of a kind of liposome loaded with CAPE significantly promotes BBB penetration and increases CAPE concentration in the ischemic brain (Lu et al., 2022). Therefore, it may be possible to improve CAPE brain targeting by investigating new NBDD.

#### 4.1.6 Sulforaphane

Sulforaphane (SFN) (1-isothiocyanato-4-methylsulfonylbutane), an aliphatic lipophilic organosulfur, is obtained from the plants of cauliflower, broccoli, and cabbage. According to previous research, SFN possesses various biological activities, such as antioxidant, anti-inflammatory, and antiapoptotic properties. Given that inflammatory responses and oxidative stress are considered to be the main mechanisms of CNS diseases, SFN is a promising bioactive agent for treating PD, AD, and multiple sclerosis. Clinical studies have also shown that SFN can improve cognitive and behavioral deficits in patients with Autism Spectrum Disorder (ASD) and schizophrenia (Singh and Zimmerman, 2016; Momtazmanesh et al., 2020). Besides, another clinical trial conducted in China on the treatment of SFN in AD patients is still ongoing (NCT04213391). In terms of the neuroprotective effects of SFN on chemobrain, recent research has shown that SFN-loaded within iron oxide nanoparticles (SFN-Fe_3_O_4_) via intranasal administration significantly alleviated cisplatin induced neurotoxicology (Ibrahim Fouad et al., 2022). Furthermore, treatment with SFN-Fe_3_O_4_ significantly restored GSH and NO contents as well as inhibited the levels of lipid peroxidation (LPO) in rat brain tissues (Ibrahim Fouad et al., 2022). Besides, SFN-Fe_3_O_4_ also inhibited cisplatin-induced upregulation of AChE. Interestingly, Fouad et a. found that compared with free SFN, SFN-Fe_3_O_4_ had greater neuroprotective potential in animal models, which suggests to us that NBDD-based SFN-Fe_3_O_4_ may have better brain targeting, physicochemical stability, and bioavailability. Notably, long-term exposure to Fe_3_O_4_ might increase the generation of ROS and trigger neurotoxicity. In general, brain-targeted FPN is an effective strategy for chemobrain treatment, but more nanoparticles need to be further studied by scientists.

### 4.2 Flavonoid

#### 4.2.1 Luteolin

Luteolin (LUT) (3,4,5,7-tetrahydroxy flavone) as a naturally sourced flavonoid, is abundant in a variety of plant species, including chrysanthemum flowers, broccoli, celery, and hot pepper. It is well documented that LUT can penetrate BBB by regulating Rho GTPase and exerting neuroprotective effects. Recently, Imosemi et al. reported that LUT significantly reversed DOX-induced downregulation of catalase (CAT), SOD, glutathione peroxidase(GPX), and glutathione S-transferase(GST) activities as well as upregulation of LPO and reactive oxygen and nitrogen species (RONS) levels in cerebral, cerebellar cortex and hypothalamus (Imosemi et al., 2022). Furthermore, given that DOX has been reported to induce neuroinflammation in many studies, and LUT can alleviate DOX-induced inflammatory responses by decreasing the NO and MPO levels as well as inhibiting TNF-α and IL-1β production (Imosemi et al., 2022). Interestingly, it has been reported that apoptosis induced by LUT has been regarded as one of the primary mechanistic in the treatment of breast cancer. However, LUT can also block DOX-triggered activities of caspase-3 in brain tissues, indicating that LUT can significantly inhibit neuronal cell apoptosis caused by DOX (Imosemi et al., 2022). However, the low water solubility of LUT largely minimizes its bioavailability and effectiveness (Majumdar et al., 2014). Recently, several studies highlighted the drug brain targeting properties of nanoparticles based-LUT through BNDD. A previous study verified that intranasal administration of chitosan-coated nanoemulsion containing LUT could be detected in brain tissues, which remarkably increased brain bioavailability as well as improved the half-life time of LUT in both plasma and brain (Diedrich et al., 2022). Abbas and colleagues designed a novel LUT-loaded chitosan decorated nanoparticles that could significantly improve the short-term and long-term spatial memory, reduce Aβ aggregation and hyperphosphorylated-tau, and inhibit pro-inflammatory mediators’ levels in Alzheimer’s disease models intranasally (Abbas et al., 2022). The bile-salt-based nano-vesicles loaded with LUT via intranasal administration also effectively improved the progression of AD compared to LUT suspension (Elsheikh et al., 2022). Collectively, LUT is a promising drug for the treatment of CRCI, and the delivery of LUT-loaded nano drugs through the NBDD is a new strategy.

#### 4.2.2 Quercetin

Quercetin (Que), a dietary flavonoid compound mainly from cherry, onions, tea, strawberry, and grape, possesses multiple pharmacological properties. Due to its lipophilicity features, Que can easily diffuse across the BBB, such that Que can reach the brain region and perform neuroprotective actions. *In vivo* experimental study, DOX treatment caused depression-like behavior disorders and oxidative damage by elevating MDA contents and reducing GSH and GST levels in the brain tissues of rats (Merzoug et al., 2014). Meanwhile, it has been reported that the immunosuppressive and myelosuppressive were serious adverse reactions of DOX exposure. DOX increased the plasma levels of corticosterone as well as altered hematological changes, to be precise, DOX decreased the total white blood cell numbers and the percentage of lymphocytes (Merzoug et al., 2014). Nevertheless, Que administration effectively improved behavior deficits, decreased MDA levels, up-regulated GSH and GST expression, reduced corticosterone levels in plasma, and restored leucopenia and lymphopenia in DOX-treated rats (Merzoug et al., 2014). Chemotherapy during pregnancy could lead to oxidative damage in the fetal brain, which may be the reason for congenital malformations (Cevik et al., 2013). Interestingly, Doğan et al. showed that compared with CYC- or DOX-exposed female rats, Que significantly reduced the activities of MDA and as well as up-regulated the expression of SOD, GSH, and CAT in fetal brain tissues (Dogan et al., 2015). Notably, human trials of Que have generally been well tolerated. Administration of Que at a concentration of 1000 mg/day or more for several months showed no adverse effects in serum electrolytes, renal and hepatic function, blood parameters, or hematology (Batiha et al., 2020). Ferry et al. studied the pharmacokinetic properties of Que in cancer patients intravenously, and they verified that 945 mg/m2 was a comparatively safe dose (Ferry et al., 1996). Taken together, Que may be an effectively neuroprotective agent and it is expected to act as an effective agent to treat chemobrain. However, the poor solubility and bioavailability of Que resulted in low levels in the circulatory system and organs. Consequently, it’s in great demand to develop novel dosage forms for Que to target the brain via NBDD. Ahmad and workers designed a Que-loaded mucoadhesive nanoemulsion (QMNE) and found that compared with oral and intravenous administration, intranasal administration of QMNE significantly increased its bioavailability and brain targeting in cerebral ischemic models (Ahmad et al., 2018). Dou and colleagues developed a natural phyto-antioxidant albumin nanoagent, HSA@QC nanoparticle, which encapsulated Que in human serum albumin, they found that intranasal administration of HSA@QC obviously alleviated Aβ aggregation, neuronal apoptosis, and oxidative stress, as well as synaptic damage in the brain of APP/PS1 mice (Dou et al., 2021). It is well known that cyclodextrins can be used as nasal excipients since they can solubilize lipophilic drugs and water-Insoluble drugs (Rassu et al., 2020). Papakyriakopoulou et al. prepared nasal powders which are composed of Que-cyclodextrins (methyl-β-cyclodextrin and hydroxypropyl-β-cyclodextrin), and the product significantly improved dissolution and nasal mucosa (Papakyriakopoulou et al., 2021). Besides, Que based-nanomaterials have been reported as promising nose-brain drug delivery systems including Que-loaded nanoemulsions, omega-3 nanoemulsions loaded with Que, and novel chitosan-coated-PLGA-nanoparticles (Ahmad et al., 2020; Vaz G. R. et al., 2022; Vaz G. et al., 2022). Therefore, the development of Que based on BNDD seems to be a feasible scheme for the treatment of chemobrain.

#### 4.2.3 Naringin

Naringin (Nar) is a prominent flavonone glycoside that is extensively present in tomatoes, grapefruits, and other citrus fruits. The pharmacological properties of Nar have been acknowledged, encompassing its anti-inflammatory, anti-oxidant, and antiradical activities, as well as its ability to suppress tumor effects. Recent research has demonstrated that Nar exhibits anxiolytic effects and neuroprotective actions against neurodegenerative disorders, traumatic brain injury, and cerebral ischemia in animal models. Furthermore, Nar has been shown to significantly ameliorate cisplatin-induced behavioral impairments and mitigate oxidative injury in the hippocampus. As we know, one of the main functions of cholinergic is responsible for modulating learning and memory (Chtourou et al., 2015). At the same time, Chtourou and colleagues have shown that Nar effectively alleviates cisplatin-induced upregulation of AChE activity in the hippocampus of rats, indicating that Nar could prevent the excitotoxicity induced by cisplatin (Chtourou et al., 2015). Furthermore, another study also suggested that Nar could alleviate oxidative injury, improve mitochondrial functions and decrease TNF-α and IL-1β levels in DOX-induced behavioral deficits mice (Kwatra et al., 2016). Interestingly, Kwatra et al. also found that Nar could alleviate DOX-induced depressive-like behavior and compared to sertraline, Nar showed comparable antidepressant effects. In addition, the combined regimen containing Nar and sertraline showed additive effects in the treatment of DOX-induced neurotoxicity (Kwatra et al., 2016). It gives us enlightenment that the combination with natural products regimen may new strategy in neuroprotection, which needs further research to confirm. Although Nar possesses the ability to cross the BBB and shows lesser adverse effects, its low bioavailability and fast elimination remarkably disturbed its clinical use. Therefore, NPs loaded-Nar may improve its delivery. For instance, compared with free Nar, the Nar phospholipid complex showed a longer half-life and stronger anti-oxidative abilities (Maiti et al., 2006). The Nar-cyclodextrin complex shows higher water dissolvability and thermal stability (Shulman et al., 2011). Furthermore, Nar-loaded liposome also exhibits improved dissolvability and bioavailability (Wang et al., 2017). However, to the best of our knowledge, little research about Nar on neuroprotection through brain drug delivery strategy was reported. More studies are needed to ascertain the insights into the safety and efficacy of NPs loaded-Nar in the treatment of chemobrain.

#### 4.2.4. Ellagic acid

Ellagic acid (EA) is a naturally four-ring polyphenolic compound abundant in vegetables and fruits, including pomegranate, grapes, strawberries, and nuts. EA contains a hydrophilic part with four hydroxyls and two lactones and a lipophilic part with two hydrocarbon rings. This structure facilitates EA to scavenge both superoxide and reactive nitrogen species. Therefore, the pharmacological characteristics of EA are fundamentally related to its anti-oxidative activity. There is growing evidence revealing that MDA is regarded as one of the biomarkers of lipid peroxidation involved in oxidative damage in nervous system diseases. It has been reported that EA could remarkably decrease the MDA contents and upregulate the GSH production in the brain tissues of rats induced by DOX (Rizk et al., 2017). Beyond the well-established antioxidative effects, EA is also known to have anti-inflammatory activities (Mishra and Vinayak, 2014). The production of TNF-α and the expression of iNOS were markedly decreased after EA treatment in DOX-treated brain tissues. Besides, EA also showed anti-apoptotic effects by decreasing the expression of caspase-3 (Rizk et al., 2017). To sum up, it is possible that EA could treat chemobrain by reducing oxidative stress, inflammatory responses, and apoptosis. Nevertheless, it should be mentioned that the poor solubility in water, poor absorption, and rapid elimination remarkably limited the usage of EA. Currently, researchers have designed EA-based micro- or nano-particulate systems, including microspheres (Ogawa et al., 2002), nanoparticles (Arulmozhi et al., 2013), liposomes (Stojiljkovic et al., 2019) and pH-dependent microassemblies (Barnaby et al., 2011). For instance, EA-loaded NPs are more effective than EA alone in decreasing oxidative homeostasis and alleviating Alzheimer’s disease (Harakeh et al., 2021). The EA-loaded calcium-alginate NPs are superior to free EA in ameliorating pentylenetetrazol-related experimental epileptic seizures (El-Missiry et al., 2020). Therefore, it’s attractive to concern the clinical applications of drug delivery systems loaded with EA in treating chemobrain.

#### 4.2.5 Rutin

Rutin, also named vitamin P, is a flavonol glycoside that extensively existed in natural plants. Rutin has been demonstrated to possess a full range of pharmacological properties, including anti-inflammatory, anti-oxidative, antidepressant, and anticancer activities. Previously, Rutin has been proved to alleviate DOX-induced cardiotoxicity as well as improve nephrotoxicity and reproductive toxicity induced by cisplatin (Alhoshani et al., 2017; Ma et al., 2017). Recent research demonstrated that Rutin could protect against DOX-triggered neuronal injury (Ramalingayya et al., 2017). Administration of ten cycles of DOX (2.5 mg/kg for 5 days) caused cognitive dysfunction in rats. However, co-administration with Rutin (50 mg/kg) effectively improved cognitive impairment (Ramalingayya et al., 2017). Furthermore, Rutin treatment significantly improved morphological changes caused by DOX, particularly neurite length, and width. In addition, the neuronal apoptosis, intracellular ROS, and the levels of TNF-α were remarkably decreased during Rutin treatment, suggesting that Rutin might be a good anti-chemobrain drug candidate (Ramalingayya et al., 2017). Ramalingayya and colleagues also found that Rutin treatment alleviated episodic and spatial memory impairment, improved myelosuppression, and ameliorated brain oxidative stress induced by DOX (Ramalingayya et al., 2019). Besides, they also verified that pretreatment with Rutin had little influence on the anticancer activity of DOX (Ramalingayya et al., 2019). In a word, these studies indicated that Rutin has the potential to be an effective drug for chemobrain. Additionally, the neuroprotective role of Rutin against cisplatin-induced neurotoxic was also reported. Cisplatin causes peroxidation of lipid membranes by increasing free oxygen radicals and decreasing the production of antioxidants, which ultimately leads to the death of neurons (Sugihara and Gemba, 1986). It is well known that the upregulation of TBAR in intracellular indicates the increasing of free oxygen radicals and glutathione guards against free radical assault (Slater et al., 1987; Meister, 1991). Rutin treatment improved TBAR and GSH changes in rats challenged by cisplatin (Almutairi et al., 2017). Moreover, in the brain, as anti-oxidants, paraoxonases (PONs) play a key role in nerve myelination (Tang et al., 2011). Exposure to cisplatin significantly reduced PON-1 and PON-3 expression by at least four-fold. Interestingly, Almutairi et al. have shown that administration of Rutin markedly restored PON-1 and PON-3 expression to normal levels (Almutairi et al., 2017). Besides, the antioxidant and anti-apoptotic effects of Rutin against the toxic effects of cisplatin in peripheral nerve tissue have been demonstrated (Yasar et al., 2019). Taşlı et al. also demonstrated that Rutin co-administration could alleviate the cisplatin-induced detrimental effects through suppressing inflammation and lipid peroxidation, thereby reducing histopathology in the retina and optic nerves (Yasar et al., 2019). Collectively, Rutin not only plays a neuroprotective effect in CNS but also shows a certain role in the peripheral nervous system. Consistent with other natural products, hepatic first-pass metabolism, and BBB contributes to less bioavailability of Rutin in the body. Hence, novel drug carrier systems and targeting the brain might be a promising way. For example, compared with free Rutin, Rutin-loaded mucoadhesive polymeric nanoparticles administrated via nasal delivery significantly markedly increased the Rutin concentration in the brain and had better protective effects on cerebral ischemia (Ahmad et al., 2016a). Ahmad and Collenges also designed Rutin-loaded chitosan NPs, which effectively improved drug delivery into the brain after intranasal administration (Ahmad et al., 2016b). In conclusion, Rutin-loaded NPs are promising in guarding neurons against cell death and oxidative stress during chemotherapy.

#### 4.2.6 Morin

Morin as a naturally resourced polyphenol extensively presents in the leaves, branches, fruits, and stems of numerous plants. Accumulating evidence have shown that Morin has a great ability to improve brain damage, including Parkinson’s disease, cerebral ischemia-reperfusion, and sepsis-triggered cognitive deficits (Xu X. E. et al., 2020; Khamchai et al., 2020; Ishola et al., 2022). Compared with DOX treated-only, Morin remarkably alleviated DOX-induced degenerative changes in neurons and hyperemia (Kuzu et al., 2018). Due to the double bond and the hydroxyl between C2 and C3 atoms, Morin has a high antioxidant potential (Solairaja et al., 2021). Notably, Morin can further inhibit MDA production and upregulated the levels of antioxidative enzyme activities, such as SOD, GSH, and GPx in brain tissues of DOX-treated animal models (Kuzu et al., 2018). It has been reported that during neuroinflammation, astrocytes are one of the main players (Ben Haim et al., 2015). Morin can also block DOX-induced activation of astrocytes in both gray matter and white matter of brain tissues, as well as decrease TNF-α and IL-1β levels. Meanwhile, Morin showed anti-apoptotic effects by upregulating the DOX-decreased anti-apoptotic gene Bcl-2 in the brain. Importantly, several studies have verified that Morin shows little toxicity and is well tolerated (Caselli et al., 2016). To sum up, Morin seems attractive in the treatment of chemobrain, but even better if it shows brain targeting further. However, to the best of our knowledge, few studies have revealed the potential therapeutic effects of Morin-loaded NPs, and the nasal brain delivery system gives us a new idea.

#### 4.2.7 Epicatechin

Epicatechin (EC) is one of the major catechins primarily exerted in cocoa, apples, and the leaves of the tea plant. A number of large-scale epidemiological studies have demonstrated positive relationships between the consumption of these EC-rich foods and cognitive function. Acute exposure to DOX significantly decreased body weight and increased the mortality rate of rats, however, pretreatment with EC remarkably reversed these changes. EC treatment markedly reduced the morphological changes induced by DOX (Mohamed et al., 2011). Moreover, administration of EC before DOX treatment significantly inhibited astrocytes activation, decreased the levels of TNF-α and NO as well as reduced TNF-α, NF-κB and iNOS mRNA levels in brain tissues. Therefore, EC can alleviate inflammatory response in the brain exposed to DOX. On the other hand, EC also reversed DOX-triggered oxidative stress with decreasing MDA production and elevating the expression level of catalase, SOD and GSH-Px. To sum up, EC has certain protective effects on chemobrain through anti-inflammatory and antioxidant properties. However, the long-term toxic and side effects of EC are still unknown. Interestingly, chitosan-coated-PLGA-nanoparticle exhibited noteworthy enhancements in nasal permeation and retention of catechin hydrate, rendering it a promising and secure brain-targeted delivery system for treating brain diseases (Ahmad et al., 2020). Therefore, the nanodrug via nose-brain delivery might improve brain-targeting and alleviate ADR.

#### 4.2.8 Juglanin

Juglanin (JUG) is a novel natural compound sourced from the herb of Polygonum aviculare (Liu et al., 2008). Prior research has established the significant antioxidant, anti-inflammatory, and anticancer properties of JUG (Zhao et al., 2020b). It has been reported that DOX exposure significantly induces body weight loss, triggers behavioral deficits, and results in depression-like behaviors. Experiment studies showed that treatment with JUG effectively reverses these changes induced by DOX (Wei et al., 2022). Furthermore, Wei et al. observed that the levels of SOD, GSH, and CAT were decreased, and the contents of MDA was increased in brain tissues of DOX-treated group, which were subsequently reversed by JUG treatment. These findings suggest that JUG may possess a protective effect on DOX-induced chemobrain by modulating oxidative stress. Additionally, studies also indicated the potential benefits of JUG in the alleviation of neuroinflammation via decreasing the expression of TNF-α, IL-6, IL-1β, and NF-KB. In addition, administration of JUG significantly lowered the DOX-elevated AchE activities and caspase-3 activity (Wei et al., 2022). Collectively, all these results suggested that JUG might be a potential drug candidate in the treatment of chemobrain. As we know, JUG is hydrophobic with low bioavailability, which limits its clinical application. However, there is a lack of research on the pharmacokinetic of JUG as well as JUG-loaded NPs targeting the brain.

#### 4.2.9 Galangin

Galangin (GAL; 3,5,7-trihydroxy flavone), a natural flavonoid extensively existing in galangal and honey, has many bioactive properties, including anti-inflammatory, antimicrobial, antiviral, anti-obesogenic, and antioxidant effects. It has been shown that GAL can reduce DOX-triggered cognitive deficits and anxiety-like behavior (Abd El-Aal et al., 2022). Of course, like other flavonoids, GAL can abolish DOX-induced inflammatory and oxidative stress in the brain by reducing the production of IL-1β, IL-6, and TNF-α and modulating the activities of MDA and GSH. Furthermore, El-Aal et al. showed that GAL significantly enhanced BDNF expression and mitigated DOX-provoked necroptosis in the hippocampus (Abd El-Aal et al., 2022). All these findings suggest that GAL is a promising drug for the treatment of chemobrain with good neuroprotective effects. At present, most studies focus on the biochemical changes caused by GAL in animal models and there is no direct evidence of its effectiveness in humans. Besides, despite the benefits of GAL in neuroprotection, its efficacy has been limited by weak intestinal absorption, poor bioavailability, and high first-pass metabolism. Therefore, more studies are needed to explore the benefit of NBDD in overcoming the restriction of GAL.

### 4.3 Terpenoids

#### 4.3.1 Ginsenoside

Ginsenosides, a class of triterpenoid saponin, serve as the primary active constituents of ginseng. Among these, Ginsenoside Rg1 (Rg1) emerges as a crucial pharmacological compound. Experimental evidence has shown that administering DAC intraperitoneally three times with a 2-day interval leads to a significant reduction in MEMRI signal intensities in brain regions and impairs cognitive performance. However, Rg1 co-administration (5 mg/kg/day or 10 mg/kg/day) 1 week prior to the DAC regimen for 3 weeks effectively mitigates these changes in a dose-dependent manner (Shi et al., 2019). Moreover, empirical evidence demonstrated that Rg1 protected DAC-induced chemobrain by regulating the microglial polarization as well as reducing the production of TNF-α and IL-6 (Shi et al., 2019). Additionally, Rg1 has been extensively investigated for its neuroprotective properties and has been widely employed in clinical settings with minimal adverse effects. However, prior studies have suggested that Rg1 exhibits poor absorption and rapid depletion, and lacks efficacy in traversing the BBB to attain the therapeutic concentrations in brain tissues (Sun et al., 2005; Zhou et al., 2014). Hence, the utilization of a novel drug delivery system to increase the transportation of Rg1 across the BBB appear to be a promising approach. For instance, the transferrin receptor (TfR), which is highly enriched in the endothelium of brain capillaries, may facilitate transcytosis of transferrin or antibodies against the TfR across the BBB. To target the brain and achieve improved therapeutic outcomes, nanoparticles containing digoxin and loperamide based on transferrin or antibodies against the TfR (OX26 antibody) have been employed. Shen et al. have designed poly-γ-glutamic acid and OX26 antibody-based nanoparticles to load Rg1 (PHRO). They discovered that administrating PHRO injection via tail venous could effectively penetrate the BBB, resulting in the sustained release of Rg1, which ultimately decreased the volume of cerebral infarction and enhanced recovery of neurons in rats suffering diabetes and cerebral infarction (Shen et al., 2017; Shen et al., 2019). Additionally, Rg1-loaded complex nanovesicles could also across BBB and promote angiogenesis (Shang et al., 2022). Notably, Li et al. developed self-assembled Dox@Rg1 nanoparticles that not only mitigated DOX-induced cardiotoxicity but also enhanced its antitumor efficacy (Li et al., 2021). Taken together, Rg1-based NPs hold promise as potential therapeutic agents for the treatment of CRCI and may offer simultaneous benefits of anti-cancer effects.

#### 4.3.2 Asiatic acid

Asiatic acid (AA) is a naturally occurring pentacyclic triterpene that serves as the primary bioactive constituent in the extract of the tropical herb Centella asiatica L. This plant has been recommended by pharmacopeia in China, Germany, and India for its potential to improve wound healing (Thong-On et al., 2014). AA has demonstrated a diverse range of pharmacological activities, including anti-inflammatory, antioxidant, and apoptosis-regulating properties, which may account for its therapeutic efficacy in various diseases. Impressively, AA exhibits a higher capacity to inhibit lipid peroxidation than several prominent antioxidants such as probucol, ascorbic acid, and alpha-tocopherol. Importantly, the lipophilicity, physicochemical properties, and bioavailability of AA suggest that AA has the potential to cross the BBB and provide neuroprotection. Previously, AA has been patented as a cognition enhancer for the treatment of dementia and amelioration of cognitive, cerebrovascular, and central nervous system conditions (EP0383171A2). with regard to mitigating chemobrain, rats exposed to 5-FU (25 mg/kg) on day 8, 11, 14, 17 and 20 exhibited cognitive impairment and accelerated cell death in the hippocampus. However, both co-treatment with AA (30 mg/kg), either before exposure to 5-FU (preventive), after exposure to 5-FU (recovery), or throughout the duration of the experiment could counteract the cognitive deficits (Chaisawang et al., 2017). JU Welbat et al. also showed that administration of AA significantly reversed the 5-FU-caused decrease of Notch1 sex determining region Y-box 2 (SOX2), nestin, doublecortin (DCX), and Nrf2 levels. Besides, AA treatment also reduced p21-positive cell number and MDA content in the hippocampus (Welbat et al., 2018). Taken together, combining the outstanding efficacy and safety properties of AA and its brain penetration ability, AA looks to be a viable drug candidate for the treatment of chemobrain.

#### 4.3.3 Ganoderic acid

Ganoderic acid (GA) is a prominent triterpenoid compound isolated from Ganoderma lucidum. A growing body of evidence shows that GA exhibits a diverse biological activity, such as antibacterial, antitumor, anti-oxidation and anti-apoptosis effects. Experimental findings indicate that the GA administration significantly improves cognitive dysfunctions in mice exposed to 5-FU, whereas both spatial and non-spatial memory decline in the absence of GA (Abulizi et al., 2021). Furthermore, studies have shown that GA confers protection against 5-FU-induced structural injuries of neurons by mitigating mitochondrial impairment, which involves ameliorating intracellular mitochondrial swelling and crest fractures, promoting mitochondrial biogenesis, and inhibiting mitochondrial fission (Abulizi et al., 2021). Pro-inflammatory cytokines have been found to exist remarkable toxic effects on neuronal cells, particularly in the hippocampus (Wang X. M. et al., 2015). Additionally, 5-FU has been shown to induce age-associated cognitive decline in mice by upregulating cytokine expression (Groves et al., 2017). Notably, supplementation of GA significantly downregulated the production of IL-6 and decreased the expression of COX-2. Biomedical studies have shown that GA is a promising drug candidate for treating chemobrain, whereas the low abundance, complex extraction, and purification procedures cause significant restrictions on its use on a laboratory scale. Hence, the challenge of augmenting the concentration of GA and enhancing the techniques for extracting and refining GA from Ganoderma lucidum necessitates resolution (Xia et al., 2014).

#### 4.3.4 Astaxanthin

Astaxanthin (AST), a xanthophyll carotenoid present in diverse microorganisms and marine organisms, possesses distinctive chemical properties due to its molecular structure. AST can directly penetrate cells and neutralize active oxygen and free radicals, thereby acting as a naturally occurring antioxidant with antioxidative activity surpassing that of vitamin E by 500 times (Wu L. et al., 2020). Prior studies have documented the diverse biological properties of AST, including immunomodulatory, antioxidant, hypoglycaemic, and hypolipidaemic activities. With regard to neurological diseases, the lipid-soluble capacity of AST enables it to readily traverse BBB and exert direct effects on CNS. Additionally, following single and repeated dietary ingestion, AST accumulates in the hippocampus and cerebral cortexes of rat brains, underscoring its importance in the treatment of neurological conditions (Zhang et al., 2014; Manabe et al., 2018). *In vivo* experimental investigations have demonstrated that AST treatment (at a dose of 25 mg/kg) confers significant protection against DOX-induced memory impairment in rats. The restorative effects of AST on hippocampal histopathological architecture and decrease hippocampal apoptotic markers (cytochrome c and caspase-3 activities) have been demonstrated (El-Agamy et al., 2018). Furthermore, AST has been reported to reduce MDA levels and increase GSH and catalase contents, while inhibiting astrocytes activation and downregulating TNF-α, PGE2, and COX-2 levels. The findings suggest that AST can strengthen the antioxidant defense system and mitigate neuroinflammation induced by DOX (El-Agamy et al., 2018). Currently, AST is widely utilized as a food additive, dietary supplement, and clinical drug with no observed side effects in animals and humans. For instance, a prior study has confirmed that there are no adverse effects observed after administrating AST (6 mg/day) in adult humans (Spiller and Dewell, 2003). Hence, it appears that minimal constraints exist in the management of chemobrain. Nonetheless, the inadequate stability and solubility of AST contribute to poor absorption, which ultimately impedes its clinical utilization. In recent times, Santonocito et al. have devised AST-loaded stealth lipid nanoparticles (AST-SSLNs) which have demonstrated a substantial improvement in the stability and bioavailability of AST in the brain. Furthermore, AST-SSLN has shown a superior antioxidant capacity in comparison to free AST (Santonocito et al., 2021). Simultaneously, Fe_3_O_4_/AST/Transferrin NPs, the AST nanoparticles coated with transferrin and polyethylene glycol (PEG), exhibits better water dispersibility and biocompatibility than free AST, resulting in heightened neuroprotective activity (You et al., 2019). Besides, the ASX thermosreversible nasal gel (ATX-NLC *in situ* gel) formulated by incorporating poloxamer-127 to AST nanostructured liposomes, can augment AST accumulation in the brain, thereby serving as an adjunctive therapeutic agent for Parkinson’s disease (Gautam et al., 2021). In general, AST represents a promising approach for the treatment of chemobrain, and nanoparticle-based delivery of AST via NBDD holds significant potential.

### 4.4 Others

#### 4.4.1 CI protein

C. indicus protein (CI protein), a 43 kD protein, is extracted and purified from the leaves of Cajanus indicus L. Historically, these leaves have been utilized for treating various hepatic disorders, such as jaundice and hepatomegaly (Manna et al., 2007). Recent research has demonstrated the protective properties of CI protein against carbon tetrachloride-related hepatic disorders, galactosamine-induced nephrotoxicity, and doxorubicin-triggered nephrotoxicity (Ghosh et al., 2006; Sinha et al., 2007; Pal and Sil, 2012). Additionally, Ghosh et al. (2006) have confirmed the capacity of CI protein in mitigating oxidant and scavenging free radicals. As we know, the overproduction of lipid peroxidation and subsequent neuronal cell death are primary indicators of neurotic injury in chemobrain. Interestingly, experimental studies have observed that CI protein administration (3 mg/kg) for 4 days markedly inhibits DOX-induced oxidative stress, reflected by decreased content of MDA, protein carbonyl and GICSSG, and upregulated-GR, GSH, and GPx activities in the brain tissue (Pal et al., 2012). The lipid-dependent ATPases that are bound to the membrane play a significant part in nerve transmission, active transport, and the maintenance of cellular homeostasis. Furthermore, CI protein treatment significantly reversed the DOX-induced decrease in the activities of Na+, K+-ATPase, Ca2+-ATPase, and Mg2+-ATPase (Pal et al., 2012). Additionally, the present study has also suggested that CI protein inhibits mitochondria-dependent apoptotic cell death by decreasing the expression of cytochrome c, caspase-3, caspase-9, Bad/Bcl-2, and PARP in the brain tissues of mice (Pal et al., 2012). These findings suggest that CI protein has the potential to prevent chemobrain and develop drugs via further research.

#### 4.4.2 C-phycocyanin

C-phycocyanin (C-PC) a protein-binding pigment that existed in cyanobacteria, is extensively utilized as a dietary supplement, colorant, and fluorescent dye for the ability to aid in the process of photosynthesis. Pharmacological studies have shown that C-PC possesses numerous biological functions, including anti-inflammatory, antioxidant, anti-necroptosis, and anti-tumor properties. Recently, C-PC has garnered significant attention in the field of neuroprotection. It has been reported that C-PC can improve multiple sclerosis by inhibiting inflammatory responses and oxidative stress (Penton-Rol et al., 2016). Additionally, C-PC has been found to exert beneficial effects on streptozotocin-induced cognitive deficits by attenuating neuroinflammation and apoptosis (Agrawal et al., 2020). Interestingly, it has also been observed that C-PC possesses neuroprotective effects on DOX-induced chemobrain. Wang et al. demonstrated that C-PC (50 mg/kg) treatment improved spatial learning and rescued synaptic density induced by DOX (Wang et al., 2021). Co-administration of C-PC with DOX significantly ameliorated mitochondrial damage in the hippocampus. Moreover, C-PC inhibited DOX-induced neuroinflammatory response in the hippocampus, as evidenced by the decrease of TNF-α, IL-1β, and IL-6 levels, as well as a reduction of the number of IBA-1 and glial fibrillary acidic protein (GFAP)-positive cells. Additionally, C-PC administration also decreased the content of MDA, protein carbonyl, and 8-OHdG while increasing the activities of GSH and SOD. Mitochondrial dysfunction has been demonstrated to participate in DOX-induced brain injury and serve as a contributing factor to CRCI. Administration of C-PC significantly ameliorated DOX-induced mitochondrial damage in the hippocampus, demonstrated by restored mitochondrial enzyme complex activity, increased mitochondrial respiratory control ratio, ATP production, and reduced ROS production (Wang et al., 2021). Importantly, as a natural constituent, C-PC has been approved to use as functional food or dietary supplement due to its nontoxic and non-carcinogenic (Romay and Gonzalez, 2000; Penton-Rol et al., 2021). Taken together, these findings suggest that C-PC is a viable candidate for the treatment of CRCI. Excitingly, the therapy for neurological diseases utilizing C-PC and BNDD has been reported with promising results. Min et al. have found that C-PC-based liposomes with 20% cholesterol administrated via the nose-to-brain system showed extended neuroprotective effects in animal models of cerebral ischemia (Min et al., 2017). Additionally, Chung et al. designed chitosan-coated C-PC liposome (C-PC liposome) and verified that nasal administration of C-PC liposome resulted in smaller infarct size and improved behavioral test outcomes in rat Middle Cerebral Artery Occlusion (MCAO) models in comparison to free C-PC (Chung et al., 2018). Collectively, these findings suggest that C-PC is a valuable candidate for treating CRCI, and its efficacy might be further enhanced by NBDD.

#### 4.4.3 Astragali Radix

Astragali Radix (AR), a traditional Chinses medicine (Huangqi), is the dried root of Astragalus species (Li et al., 2019). To date, there are over 100 compounds extracted and identified in AR, containing flavonoids, polysaccharides, saponins, and amino acids (Auyeung et al., 2016; Liu et al., 2017). Currently, it has been demonstrated that AR may offer protection against heart, lung, liver, kidney, and brain injury in a variety of oxidative stress-related disease models (Hong et al., 1992; Shahzad et al., 2016). AR administration significantly reversed DOX-induced weight loss in rat models. Furthermore, AR treatment remarkably recovered the elevation of MDA and the ratio of SOD/MDA in brain tissues caused by DOX (Yu et al., 2021). The homeostasis of amino acid metabolism is closely associated with the redox balance in the brain of mammals. Yu et al. found that DOX exposure significantly upregulated six amino acid expressions in brain tissues, including glycine, serine, glutamate, citrulline, ornithine, and alanine, which were closely associated with reactive oxygen species occurrence. Notably, the AR administration effectively reversed these changes (Yu et al., 2021). In a word, given the extremely low toxicity, AR has been approved as a dietary supplement in China and the USA, suggesting that AR is one of the most promising candidates for the treatment of CRCI. However, similar to many natural products, some compounds in AR exhibit low water solubility and inadequate bioavailability, thereby restricting their practical applications. Consequently, additional studies are necessary to enhance its utilization by optimizing its dosage form. For instance, Yu et al. made a commendable effort to combine Astragalus polysaccharide (APS) with gels to attain sustained APS release to regulate immunity (Yu et al., 2017). Yakubogullari et al. combined Astragaloside VII (AST VII) with APS to formulate nano-preparations, which served as an adjuvant of influenza vaccine, resulting in prominent regulatory effects in immunity and viral defense system (Yakubogullari et al., 2019). Hence, future research should concentrate on the dosage forms by utilizing and developing polysaccharides, flavonoids, and saponins in AR, extremely nano-preparations.

#### 4.4.4 Extract of Tiliacora triandra

Tiliacora triandra (Colebr.) Diels (TT) as a plant of the Menispermaceae family is widely spread in Southeast Asia. Due to its richness in polyphenols and flavonoid compounds, TT is commonly used for anti-pyretic, anti-bacterial, and anti-malarial activities. A prior study has explored the neuroprotection of TT and pointed to the protection of TT against brain oxidative stress, neurological degeneration, and cerebral infarction in a murine model of cerebral ischemia-reperfusion (Thong-Asa and Bullangpoti, 2020). In addition, T. triandra leaf extract has been found to possess the ability to improve spatial learning and memory and maintained ChAT activity in the hippocampus (Wachiryah and Hathaipat, 2018). Recent studies have found that TT administration (250 mg/kg or 500 mg/kg) orally for 5 weeks significantly prevents weight loss, alleviates pathological damage in the brain, and improves cognitive dysfunction induced by cisplatin (Huang et al., 2021). Simultaneously, TT significantly inhibited CDDP-induced oxidative stress and neuroinflammation by decreasing MDA levels, elevating the activities of GPx, SOD, GSH, and CAT, and impeding the production of TNF-α, IL-1β, and IL-6 (Huang et al., 2021). Bcl-2 and the caspase families are key regulators of the apoptotic pathway. It has also been reported that TT reduces acetylcholinesterase activities, decreases caspase-3 levels, and increases Bcl-2 expression in brain tissues, indicating that TT can protect against cisplatin-induced neuronal death in rat models. Collectively, although the components of the extract of Tiliacora triandra require further elucidation, the crude TT exhibits excellent potential for advancement through nanotechnology as medicine and/or nutraceutical products, given the overall efficacy against CRCI.

#### 4.4.5 Ergothioneine

Ergothioneine (EGT), a naturally occurring compound initially discovered from ergot fungus, is exclusively produced by certain fungi and bacteria. Despite the inability of animals to synthesize EGT, it is widely absorbed and transported into cells by the cell membrane-specific carnitine/organic cation transporter (OCTN1) (Grundemann, 2012). EGT is a potent physiological antioxidant, thereby exerting a protective role in many oxidative stress-related diseases, including diabetes, chronic kidney and liver diseases, diabetes, cardiovascular diseases, and cancer (Paul, 2022). Notably, EGT shows high blood-brain barrier permeability and possesses neuroprotective effects, which are associated with the expression of OCTN1 (Nakamichi et al., 2012). The regulation of cellular functions by EGT has been found in neurons, neural stem cells, microglia, and cerebral vascular endothelial cells (Ishimoto and Kato, 2022; Nakamichi et al., 2022). Recent experimental studies have shown that supplementation of EGT (2 or 8 mg/kg/day) for 58 days significantly restores EGT levels in brain tissues, prevents memory and learning impairment, and alleviates body weight loss induced by cisplatin (Song et al., 2010). Simultaneously, EGT has been demonstrated to inhibit cisplatin-triggered neurotoxicology by reducing MDA levels and increasing the ratio of GSH/GSSG (Song et al., 2010). Furthermore, *in vitro* studies have defined that EGT treatment can alleviate the cisplatin-induced damage in PC12 cell proliferation and PCN cell axonal and dendritic growth. These findings suggest that EGT prevents cisplatin-caused neuronal injury and improves cognition, which is possibly mediated by its inhibition of oxidative stress and restoration of AChE content in neuronal cells. In view of the fact that EGT can reach millimolar levels in brain tissues with little toxicity, more experimental and clinical studies on the treatment of CRCI with EGT supplements are strongly encouraged (Halliwell et al., 2018; Tang et al., 2018).

#### 4.4.6 Bixin

Bixin, a liposoluble di-apocarotenoid, is extracted from the seeds of Bixa orellana which is extensively utilized for the treatment of infectious and inflammatory diseases in Mexico and South America. Due to its safety, Bixin is certified by the FDA as a natural food colorant and additive due to its safety (Xue et al., 2018). Pharmacological studies have reported that Bixin exhibits a diverse range of activities, including anti-inflammatory, antioxidative, and anti-tumor properties (Rivera-Madrid et al., 2016). Previously, a vitro study has indicated that Bixin can capture ROS, while animal studies also demonstrated that Bixin protects against oxidative DNA damage and lipid peroxidation (Kumar et al., 2018). Furthermore, the prior investigation has established Bixin as a novel inducer of Nrf2, which has the potential to quench ROS and impede inflammation and fibrosis in lung tissue (Xue et al., 2018). Considering the correlation between chemotherapeutic drug-induced neurotoxicity and ROS production, Bixin may serve as a promising effective agent against CRCI. Experimental findings have shown Bixin markedly decreases DNA percentage in tail and micronuclei frequency in PC12 cells exposed to cisplatin. Although studies have demonstrated that Bixin can cross BBB, there is still a lack of *in vivo* studies of Bixin on CRCI (Yu et al., 2020). Therefore, more effects are still required to fully elucidate the neuroprotective effects of Bixin on CRCI and its potential mechanisms.

## 5 Regulated mechanism of natural products in CRCI

### 5.1 Keap1-Nrf2 signaling pathway

**FIGURE 2:**
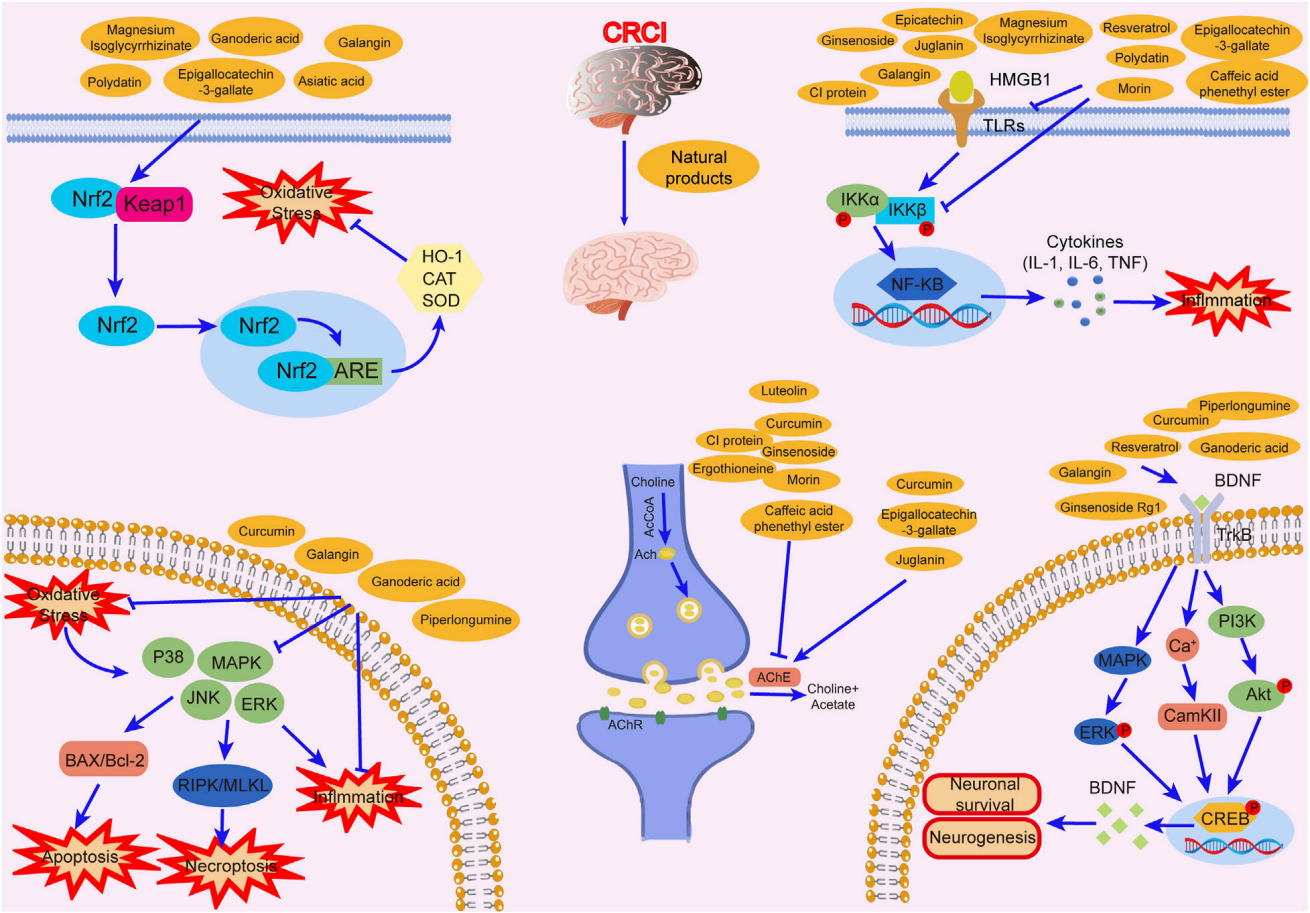
The signaling pathway of the natural products in the treatment of CRCI. The arrow symbol indicates the promoting role and the “┴” symbol indicates the inhibiting role.

The transcription factor Nrf2 (NF-E2 related factor 2), is a protein with a leucine zipper structure, accounting for strengthening the oxidative defense system and modulating oxidative stress. Under normal conditions, Nrf2 is sequestered in the cytoplasm through formulating the complexes of Nrf2, Kelch-like ECH-associated protein 1 (Keap1), and Cul3, resulting in subsequent degradation via the ubiquitin-proteasome system (Bellezza et al., 2018). During oxidative stress, the Keap1 ubiquitination system is disrupted, leading to the translocation of Nrf2 from the cytoplasm to the nucleus. In the nucleus, Nrf2 binds to antioxidant response element (ARE) and triggers the transcription of numerous antioxidation-related genes, including NAD(P)H quinone dehydrogenase 1 (NQO1), Heme oxygenase-1 (HO-1), and glutathione peroxidase (GPx) (Yu and Xiao, 2021). Increasing evidence has shown that Nrf2 alleviates multiple stressors-induced cell damage via modulating the gene expression that counteracts oxidative stress or activates the antioxidative system (Abed et al., 2015). Currently, Keap1-Nrf2 signaling pathway has been defined to play an essential role in the treatment of CRCI. For instance, Polydatin can mitigate CRCI by upregulating Nrf2 levels and GSH production in the hippocampus (Tong et al., 2020). Furthermore, Hamed Arafa et al. has revealed that Epigallocatechin-3-gallate offers protection against cisplatin-induced cognitive impairment by enhancing the Nrf2 signaling pathway and subsequently upregulating the expression of antioxidant enzyme genes HO-1 and TAC (Arafa and Atteia, 2020). Additionally, natural products including Galangin, Asiatic acid, Ganoderic acid, Magnesium Isoglycyrrhizinate, and Piperlongumine also activate the Nrf2 signaling pathway and alleviate oxidative stress to attenuate CRCI (Welbat et al., 2018; Jiang et al., 2019; Abulizi et al., 2021; Abd El-Aal et al., 2022; Ntagwabira et al., 2022). Interestingly, recent studies have revealed that Nrf2 activation can also regulate the transcription of genes related to mitochondrial functions, neuroinflammation, autophagy, and ferroptosis (Zuo et al., 2022). Therefore, Nrf2 exerts neuroprotective roles through the regulation of multiple pathways. It’s reasonable to confirm that Nrf2 is a powerful target for the treatment of CRCI (Figure 2).

### 5.2 HMGB1/TLR4/NF-κB pathway

High mobility group box 1 (HMGB1) is a damage-associated molecular pattern molecule exerting function via binding to cell surface receptors, such as Toll-like receptor 4(TLR4) (Kang et al., 2014). When cells are stimulated, HMGB1 in the nucleus will translocate to the cytoplasm to bind with TLR4 and subsequently activate NF-κB, resulting in enhanced production of inflammatory cytokines, such as TNF-α, IL-1β, and IL-6 (Liang et al., 2020). It has been defined that HMGB1/TLR4/NF-κB signaling pathway plays a significant part in promoting inflammatory responses (Xu X. et al., 2020). Notably, the NF-κB signaling pathway can modulate diverse biological functions, including inflammation, immunity, apoptosis, cell stress response, and cell adhesion (Jing and Lee, 2014). Thus, blockade of HMGB1/TLR4/NF-κB signaling pathway activation-enhanced inflammatory responses may be a powerful pharmacological intervention for the treatment of CRCI. Studies have demonstrated that Galangin can inhibit the HMGB1/TLR4/NF-κB signaling pathway and decrease the production of TNF-α, IL-1β, and IL-6 (Abd El-Aal et al., 2022). Similarly, CI protein, Magnesium Isoglycyrrhizinate, Juglanin, Epicatechin, Morin, Caffeic acid phenethyl ester, Epigallocatechin Gallate, and Polydatin also alleviated neuroinflammation via blocking TLR4/NF-κB signaling (Mohamed et al., 2011; Pal et al., 2012; Kuzu et al., 2018; Jiang et al., 2019; Ali et al., 2020; Arafa and Atteia, 2020; Tong et al., 2020; Wei et al., 2022). Additionally, Resveratrol and Ginsenoside Rg1 inhibit inflammatory responses through inhibiting PPARγ/NF-κB signaling pathway (Shi et al., 2018; Shi et al., 2019). Hence, targeting HMGB1/TLR4/NF-κB or PPARγ/NF-κB signaling pathway and then inhibited the production of inflammatory cytokines might be a promising strategy treating CRCI.

### 5.3 Mitogen-activated protein kinase (MAPK) pathway

MAPKs, a group of serine-threonine protein kinases, are responsible for regulating various cellular properties in response to a wide range of extracellular stimuli. Extensive research has demonstrated the crucial role of the MAPK family in modulating the proliferation, survival, differentiation, and apoptosis of cells (Sun et al., 2015). Currently, several MAPK members have been recognized, including p38 MAPK, c-jun N-terminal kinase (JNK), and extracellular signal-regulated kinase (ERK1/2) et al. Recent work has suggested that in reaction to extracellular stimuli, especially chemotherapeutic agents, MAPK can facilitate apoptosis through modulating apoptotic-related proteins such as Bcl-2, BAX, and Bim (Sui et al., 2014). Hence, targeting p38 MAPK, ERK1/2, or JNK MAPK is considered a promising strategy to prevent neuronal cell death in CRCI. It has been discovered that inhibition of cell apoptosis is involved in the protective effects of CUR against cisplatin-induced cognitive deficits (Yi et al., 2020). Necroptosis is also implicated in learning and memory impairment in various neurodegenerative diseases (Yuan et al., 2019). Galangin has been demonstrated to significantly inhibit the phosphorylation of three members of the MAPK family, namely p38MAPK, JNK1/2, and ERK1/2, and alleviate necroptosis biomarkers in the brain exposed to DOX (Abd El-Aal et al., 2022). A growing body of evidence supports that Ganoderic acid and Piperlongumine also possess the ability to alleviate CRCI through regulating MAPK signaling (Abulizi et al., 2021; Ntagwabira et al., 2022). In addition, some studies have highlighted the essential role of the MAPK signaling pathway in oxidative stress and inflammation, thus targeting MAPK might be a promising strategy to alleviate CRCI. However, the intricacies surrounding the activation of MAPK signal pathways necessitate further investigation to elucidate the mechanisms by which natural products modulate the MAPK family in the treatment of CRCI.

### 5.4 BDNF-TrkB pathway

BDNF as a 27-kDa polypeptide is recognized as a significant neurotrophic factor that plays a crucial role in cell survival, proliferation, and differentiation in both peripheral and central neurons. BDNF is widely acknowledged for its involvement in use-dependent plasticity mechanisms, such as learning, memory, and long-term potentiation (Colucci-D'Amato et al., 2020). *In vivo*, BDNF can bind to the receptor tropomyosin receptor kinase B (TrkB) and initiate a series of signaling pathways, including PI3K/Akt, MAPK/ERK, and Ca2+/calmodulin-dependent protein kinase II (CaMKII), ultimately leading to the enhanced transcription of cAMP-response element binding protein (CREB)-regulated genes (Leal et al., 2014). For instance, resveratrol treatment has been demonstrated to reverse DAC-induced downregulation of BDNF and TrkB, and overexpression of p-CaMKII in both the prefrontal cortex and hippocampus (Shi et al., 2018). Similarly, Ginsenoside Rg1 also has been found to elevate the levels of BDNF and TrkB as well as decrease p-CaMKII expression in DAC-induced CRCI models (Shi et al., 2019). Ganoderic acid has been found to have the potential in mitigating cognitive impairment induced by 5-FU through the BDNF-TrkB-ERK-CREB axis (Abulizi et al., 2021). Similarly, Galangin can also treat CRCI via modulating this signaling (Abd El-Aal et al., 2022). These studies collectively suggest that targeting the BDNF-TrkB pathway and safeguarding against neuronal cell death caused by chemotherapy may offer a therapeutic approach for CRCI. Nevertheless, it cannot be ignored that BDNF and its receptor TrkB are remarkably upregulated in a wide range of tumors (Jin et al., 2021; Malekan et al., 2023). Activation of the BDNF-TrkB signaling pathway can exert oncogenic effects, including promoting cancer cell survival and growth and decreasing chemotherapeutic sensitivity (Xu et al., 2019). Thus, further studies are required to ascertain whether is it still safe to activate BDNF-TrkB for the treatment of CRCI.

### 5.5 Cholinergic pathway

Cholinergic synapses are widespread throughout the brain. The choline and acetyl coenzyme A can produce ACh, which subsequently transports to the synapse where it interacts with cholinergic muscarinic (metabotropic) and nicotinic (ionotropic) postsynaptic receptors. AChE, a serine hydrolase, possesses the ability to terminate neuronal transmission at the cholinergic synapse through hydrolyzing ACh into choline and acetate ions (Massoulie et al., 1993). Numerous studies have indicated that dysfunction within the cholinergic system is implicated in a broad spectrum of cognitive disorders. It is important to acknowledge that numerous pathophysiological factors induce the occurrence of CRCI which have yet to be fully understood, and the cholinergic hypothesis does not serve as a causal explanation for the disease. For example, the expression and activity of AChE were significantly decreased by cisplatin exposure, while the levels of ACh were elevated in the brain cortex of rats. Epigallocatechin Gallate treatment effectively reversed these alterations and mitigated cognitive impairments (Arafa and Atteia, 2020). Consistently, Kandeil et al. also found that treatment with only CP and TQ significantly increased AChE activities in the brain tissues in cisplatin-induced CRCI models. However, A. Ali et al. Have reported that DOX treatment obviously increases the expression of AchE and downregulates the levels of Ach in both hippocampal and cortical tissues, and CAPE treatment significantly decreased AchE levels and upregulated Ach contents (Ali et al., 2020). In line with these findings, several other studies have also observed similar results in DOX-triggered CRCI models. Additionally, the protective effects of Morin, Juglanin, and CI protein against CRCI is associated with the inhibition of AchE (Pal et al., 2012; Kuzu et al., 2018; Wei et al., 2022). It is important to note that the contradictory results observed in these studies may be attributed to variations in the chemotherapy drugs and the specific method employed to determine AChE levels in different brain regions. Additionally, it is noteworthy that various types of tumors have exhibited an aberrant expression of AChE. AChE plays a pivotal role in regulating oncogenic signaling pathways which is associated with cellular proliferation, differentiation, and adhesion. Consequently, this discovery raises concerns regarding potential detrimental effects on patients, necessitating further investigation to determine the safety of activating AChE as a treatment for CRCI.

## 6 Limitations and future prospects

**FIGURE 3:**
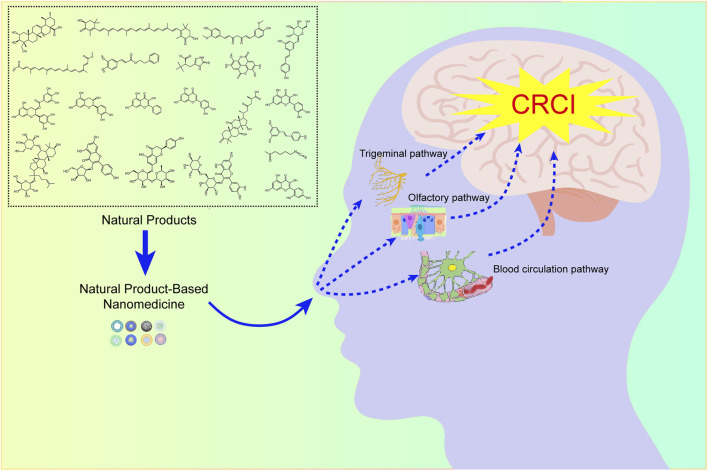
Nose-to-Brain drug delivery based natural products prospects in treating CRCI.

It is well known that natural products are a tremendous source of new drug discovery. Natural products show clearly anti-inflammatory, antioxidant, and neuroprotective effects and have attracted the growing interest of scientists. This review summarized the newest discoveries on natural products for the treatment of CRCI. As shown in numerous studies, these natural products may protect against CRCI by regulating different signaling pathways. Nevertheless, further studies are required to improve the delivery of natural products into brain tissues in the future. It is essential for researchers to study these issues thoroughly (Figure 3).

Nose-to-brain delivery offers tremendous opportunities to enhance drug translocation into brain tissues for treating CNS disorders. Administrating drugs via olfactory and trigeminal nerve pathways circumvents can avoid the disadvantages of oral administration, such as first-pass metabolism, BBB transport, and enzyme degradation, resulting in elevated-brain bioavailability, and minimized-systemic side effects. Furthermore, to further eliminate the existed-restriction of nasal administration, novel strategies, such as nanosized drug carriers have been extensively utilized, which markedly extend the drug resident time, and promote drug transportation via nasal mucosa, leading to promoted drug bioavailability and elevated therapeutic efficacy. Additionally, it has been suggested that optimizing parameters, such as phospholipid composition, PEGylation, and liposome modification, can further promote drug bioavailability in brain tissues. These drug nanoparticles based on NBDD can block drug degradation in the nasal route, providing long-term sustained release and ameliorating the conglutinative degree of nasal mucosal. Additionally, the drug nanoparticles based on NBDD are difficult to remove by the mucociliary system and therefore easily across the BBB due to their specific size and physiochemical properties.

However, there are existing several potential disadvantages of delivering drug-loaded nanoformulations into brain tissues through nose-to-brain delivery systems. Although the utilization of intranasal administration can benefit to some extent, the absorption rate and membrane permeability of natural products, which are found to be helpful for treating CRCI, are still low. Consequently, there is a necessity to promote drug absorption in nasal mucosa through adopting osmotic enhancers or new dosage forms. Notably, some agents may possess nasal mucosa toxicity, thus, the toxicity evaluation is needed before intranasal administration. And the nasal drug preparations should be taken seriously and comprehensively. Naturally sourced bioactive agents are precious resources for new drug discovery in the treatment of brain diseases, and NBDD is beneficial for elevating the absorption, and bioavailabilities of these natural agents in treating brain diseases. However, it has been discovered that some drug-loaded lipids or polymers show brain toxicity. Thus, more researches are required to design novel biodegradable materials which are safe and can be metabolized in the brain. Meanwhile, the NBDD delivery system also possesses limitations, for example, drug degradation, mucociliary clearance, and less residence time in the nasal cavity.

## 7 Conclusion

Collectively, accumulating research has pointed out that natural products have potential benefits for treating CRCI. Nevertheless, how to decrease the toxicity and elevate the bioavailability of natural products is a critical challenge. NBDD is believed to be able to promote effectiveness and mitigate toxicity for treating CRCI. Therefore, more in-depth research is required to make great progress in the utilization of NBDD in delivering natural products into brain tissues for treating CRCI.
